# Snotwatch: an ecological analysis of the relationship between febrile seizures and respiratory virus activity

**DOI:** 10.1186/s12887-022-03222-4

**Published:** 2022-06-22

**Authors:** Rana Sawires, Martin Kuldorff, Michael Fahey, Hazel Clothier, Jim Buttery

**Affiliations:** 1 Department of Paediatrics, Faculty of Medicine, Nursing and Health Sciences, Monash University, Clayton, Victoria Australia; 2 Murdoch Children’s Research Institute, Royal Children’s Hospital, Parkville, Victoria Australia; 3 Division of Pharmacoepidemiology and Pharmacoeconomics, Department of Medicine, Harvard Medical School and Brigham and Women’s Hospital, Boston, MA USA; 4 Department of Neurology, Monash Children’s Hospital, Clayton, Victoria Australia; 5 Neurogenetics Department, Monash Paediatrics, Monash University, Clayton, Victoria Australia; 6 School of Population & Global health, University of Melbourne, Parkville, Victoria Australia; 7 Child Health Informatics, Department of Paediatrics, University of Melbourne, Parkville, Victoria Australia

## Abstract

**Background:**

Febrile seizures are the commonest type of seizure in occurring in the first few years of life, mostly affecting children aged six months to five years old. While largely benign, the incidence of each febrile seizure increases the risk of recurrence, afebrile seizures and epilepsy. Viruses are the most frequent cause of febrile illnesses in which a febrile seizure occurs. Febrile seizure presentation patterns appear to follow a seasonal trend.

**Aims:**

To identify patterns of febrile seizure incidence across different seasons with specific viral activity, and to establish a framework for analysing virus circulation data with common illnesses within a shared region and population.

**Setting:**

Our study was a study of febrile seizure presentations in Victoria, Australia and respiratory virus detection.

**Participants:**

We obtained independent datasets of emergency department febrile seizure presentations at Monash Health and all respiratory multiplex PCR tests performed at Monash Health from January 2010–December 2019 to observe common trends in virus circulation and febrile seizure incidence.

**Study design:**

Trends were studied temporally through mixed effects Poisson regression analysis of the monthly incidence of febrile seizures and the rate of positive PCR tests. Peak viral seasons (95th centile incidence) were compared to median viral circulation (50th centile incidence) to calculate peak season risk ratios.

**Results:**

We found a 1.75–2.06 annual risk ratio of febrile seizure incidence in June–September. Temporal analysis of our data showed this peak in febrile seizures was attributable to circulating viruses in this season, and virus modelling showed correlation with increased rates of positive Influenza A (1.48 peak season risk ratio), Influenza B (1.31 peak season risk ratio), Human metapneumovirus (1.19 peak season risk ratio) and Respiratory Syncytial Virus (1.53 peak season risk ratio) on PCR testing.

**Conclusion:**

Our ecological study statistically demonstrates the recognised winter peak in febrile seizure incidence and ascribes the seasonal relationship to several viral infections which affect the community, including a novel association with Human metapneumovirus.

**Supplementary Information:**

The online version contains supplementary material available at 10.1186/s12887-022-03222-4.

## Introduction

Febrile seizures are the most common cause of seizure in childhood, with an incidence of 2–5% in North America and Europe [1–4]. They occur in the presence of a fever (> 38 °C) in children aged typically between 6 months and 5 years [5], where the seizure is not caused by an underlying central nervous system (CNS) infection or metabolic disturbance [5, 6]. Simple febrile seizures are single, generalised convulsions lasting less than 15 min. Complex febrile seizures comprise approximately 20–35% of all febrile seizures [7–10] and present with focal features, occur as clusters of episodes during a 24-h period or those that last for 15 min or more [5].

Viral infections are well-described as the predominant causative agents in febrile seizures, being detected in up to 82% of children with febrile seizures [10–14], although bacterial infections and vaccinations have been implicated in some cases [10, 15]. Further, the seasonality of febrile seizures, which peaks in fall and winter [16–18], supports their association with spikes in the incidence of upper respiratory tract infections (URTIs) and their causative viruses [3, 4, 13, 19–21]. Specific viruses have previously been implicated as the cause of febrile seizures, including Human Herpesvirus-6 (HHV-6) [22], Influenza A and B [20, 23], Respiratory syncytial virus (RSV) [24, 25], Parainfluenza [10, 13], Adenovirus [10, 26], Rhinovirus [11, 13] and Enterovirus [20].

Recent innovations in viral respiratory molecular diagnostics allow multiple viruses to be tested simultaneously using multiplex polymerase chain reaction (PCR) [27].

Given the known seasonal trends in prevalence for many common childhood viruses [13, 20, 23, 28–30], viral PCR data may be collated as ecologic data to track temporal viral activity in the community and explore the relationship of febrile seizures with common viral infections.

This study examined the temporal associations between organism epidemiology and health outcomes across south-eastern Melbourne. We aimed to identify patterns of febrile seizure incidence across different seasons with specific viral activity and provide insight into the impact of specific viruses on the frequency and severity of febrile seizure presentations. This technique potentially allows evaluation of the relationship between virus circulation and the burden of febrile seizures on the health system. These findings can, in turn, inform models addressing impact of therapies and vaccines against key viruses.

## Methods

### Study design and cohort

We conducted a retrospective ecologic cohort analysis of 10 years of data from 2010 to 2019. Independent, unlinked datasets from the same large hospital network health care provider were used to visualise and define associations between febrile seizures and virus activity. Monash Health is the largest health network in Victoria, Australia, based in south-east Melbourne, with three emergency departments caring for children and adults with more than 210,000 presentations annually [31]. Our inclusion criteria were all respiratory multiplex PCR assays from patients of all ages performed at Monash Health, and all febrile seizure presentations to Monash Health emergency departments recorded between 1st January 2010 and 31st December 2019. No patient records were available for analysis for patient de-identification. Therefore, the length of stay in the emergency department was used as a surrogate measure for the severity of the febrile seizure- febrile seizures with a stay of more than 4 hours duration were classified as “severe”.

Our exclusion criteria were febrile seizure presentations for patients > 5 years old as febrile seizures are not diagnosed in children beyond this age [8].

### Data collection

Emergency department (ED) febrile seizure presentation data were extracted from Monash Health business intelligence portal. Emergency department admissions with discharge diagnoses coded for febrile seizures (ICD-10-AM code R56.0) were obtained. Data extracted included: the patient’s age in years, gender, date of presentation and the length of stay in hours. Data were de-identified.

Respiratory multiplex PCR data were extracted through Monash Health pathology from the Medipath® system (LRS Health, Melbourne, Australia). This dataset included all respiratory PCRs performed from January 2010 to December 2019, irrespective of the result. Extracted data included: date of the test; the patient unit record number (URN); date of birth, gender, sampling site, and viruses detected on PCR. Data were de-identified before analysis.

Viruses included in the PCR assay were Influenza A and B, Parainfluenza 1, 2 and 3, Human metapneumovirus (hMPV), RSV, Adenovirus and Picornavirus.

### Statistical analysis

The coding program, R (version 4.0.2) [32], was applied through RStudio (version 1.2.5) [33] for statistical analysis of temporal data. To account for the significant increase in the number of total PCR tests performed each year at our health service, viral incidence data were converted into a monthly rate.

We created two different models for our datasets, applying a mixed effect Poisson regression technique. The first model used the month of the year (MOY) as an independent predictor of febrile seizures. This model enabled us to determine the trend in febrile seizure presentations throughout the year. All risk ratios (RR) in the MOY model were relative to the average number of febrile seizures in April. We chose April as the reference month because in Victoria, it coincides with the middle of fall and was thus an appropriate centre between Winter and Summer.

The second model was a multivariate model, using all viruses as predictors of febrile seizures. This model enabled us to ascertain which viruses were related to febrile seizure presentations, thus explaining the observed seasonal trends. For each virus, in any given month, the number of positive tests was divided by the total number of PCR tests performed during that month as per the following formula:

$$Monthly\ virus\ proportion\ for\ each\ virus=\frac{Monthly\ positive\ PCR\ tests\ for\ each\ virus}{\ Monthly\ total\ PCR\ tests\ performed}$$

We modelled the month of year and viruses separately due to their collinearity; that is, months which were more strongly associated with febrile seizure presentations were likely associated due to higher levels of virus circulation in those months.

The MOY and virus models were followed by subgroup analysis, where models were created to explain the number of mild and severe febrile seizure presentations, and the age groups (0–1 years, 1–2 years, 3–5 years) in which febrile seizures presented.

We aimed to compare the influence of peak virus circulation on febrile seizure presentations with the median expected virus circulation. Thus, we compared the risk of febrile seizures when viruses were present at or above the 95th percentile of their maximum rate to the 50th centile of their maximum rate. Our significance level was set at *p* <  0.01.

### Ethics approval

As per our study protocol (Appendix A), a waiver of informed consent was sought, as all patient data was de-identified and could not be traced back to the patients. All methods for this ecological study were carried out in accordance with the relevant guidelines and regulations.

The informed consent waiver was provided by Monash Health Human Research Ethics Low Risk Panel, our primary Human Research Ethics Committee (HREC). This was obtained beginning 24th July 2019 (NMA/ERM Reference Number: RES-19-0000333 L-53611). Ethical approval through our primary Human Research Ethics Committee (HREC) was obtained in Monash Health from 24th July 2019 (NMA/ERM Reference Number: RES-19-0000333 L-53611). See Appendix A.

## Results

### Cohort demographics

#### Febrile seizure cohort

There were a total of 4836 emergency department febrile seizure presentations over the study period in children aged 5 years or younger, equating to approximately 40.3 presentations of febrile seizures per month. Almost half (46.3%) of all presentations were in one-year-old (12–23 months) children and 24.6% in two-year-old (24–35 months) children (see Appendix B.- Fig. 4 For the age distribution of febrile seizure presentations).

Mild febrile seizure presentations (length of stay < 4 h) accounted for 78.1% of our cohort. Severe presentations were more frequent in children under 1 year.

#### Respiratory multiplex PCR cohort

A total of 93,873 respiratory PCR tests were performed. The number of tests performed each year increased substantially from 2010 to 2019 (Appendix C, Table 3). However, the annual proportion of viruses detected remained approximately constant throughout the study period. The majority of multiplex PCR assays (63%) were negative for all viruses tested, 26% detected one virus and 11% detected 2 or more viruses.

### Data visualisation

**Fig. 1:**
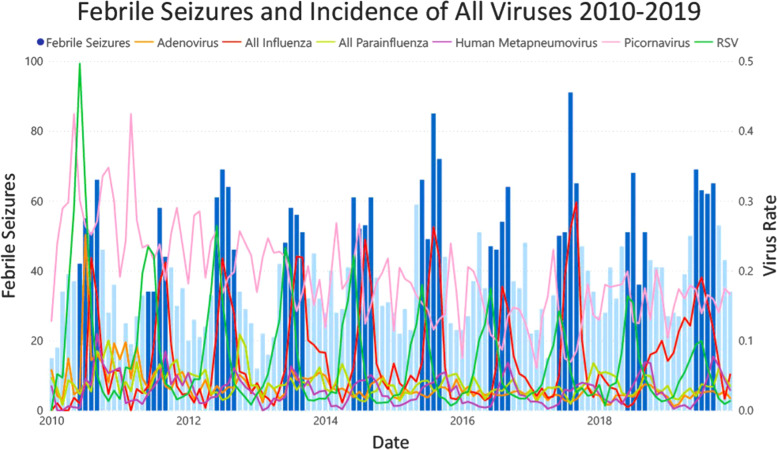
Febrile seizure presentations and virus rate by month of year

Many viruses demonstrated seasonal patterns with winter peaks including RSV, Influenza and hMPV (Fig. 1). A higher rate of positive PCR assays was observed in 2010 and 2011 compared with subsequent years. A clear winter peak was only observed in febrile seizures occurring in children aged 1–2 years old, with a less clear trend in 3-year-old children, and no distinguishable trends at the extremes of our cohort (in children less than one, and in 5-year-old children). (Appendix D, Fig. 5).

### Febrile seizure association with season

More febrile seizure presentations occurred in May to October compared to April. The greatest risk was in the month of August where there was a 2.03 (99% CI 1.70–2.44) risk ratio of febrile seizure incidence. Furthermore, January showed a reduced risk of febrile seizure presentations (RR 0.72, 99% CI 0.57, 0.91). The complete results of the monthly model are shown in Table 1.

**Table 1:** Risk of ed. febrile seizure presentations by month of year

Month	Risk Ratio	99% CI	*P*-value
January^*^	0.72	0.57, 0.91	0.0002
February	0.86	0.69, 1.07	0.08
March	0.94	0.76, 1.16	0.46
April (Reference)	1.00	–	–
May^*^	1.37	1.12, 1.66	< 0.0001
June^*^	1.76	1.46, 2.12	< 0.0001
July^*^	1.82	1.51, 2.19	< 0.0001
August^*^	2.03	1.70, 2.44	< 0.0001
September^*^	1.95	1.62, 2.34	< 0.0001
October^*^	1.38	1.14, 1.68	< 0.0001
November	1.15	0.94, 1.41	0.071
December	1.13	0.92, 1.39	0.12

This table shows the relative risk of febrile seizure presentations compared with April. At baseline, the Poisson model estimates 29.9 febrile seizure presentations in April, which was our reference month. Months with statistically significant risk ratios are denoted with^*^

The monthly model showed the same increase in febrile seizures between May and October across all subgroups, except in children less than 1 year old.

### Febrile seizure associations with viral circulation

Influenza A and B, hMPV and RSV were significantly associated with changes in febrile seizure incidence. The results of our model for the relationship between viruses and febrile seizures shown in Table 2.

**Table 2:** Risk of ed. febrile seizure presentations associated with respiratory viruses

Virus	95% vs 50% Incidence Risk	99% CI	*P*-value
Adenovirus	1.00	0.93, 1.08	0.98
hMPV*	1.19	1.07, 1.33	< 0.0001
Influenza A*	1.48	1.32, 1.67	< 0.0001
Influenza B*	1.31	1.18, 1.46	< 0.0001
Parainfluenza 1	0.94	0.79, 1.12	0.35
Parainfluenza 2	0.94	0.85, 1.05	0.14
Parainfluenza 3	1.06	0.94, 1.19	0.25
Picornavirus	1.04	0.92, 1.19	0.39
RSV*	1.53	1.38, 1.70	< 0.0001

Relative risk of febrile seizure incidence compared to a baseline incidence of 21.1 per month. Statistically significant risk ratios (*p* < 0.01) are denoted with *

Subgroup analysis did not yield any notable differences to the all-group febrile seizures analysis. The complete tables for the subgroup virus models are in Appendix E (Tables 4, 5, 6, 7, 8, 9, 10, 11, 12, 13).

## Discussion

**Fig. 2:**
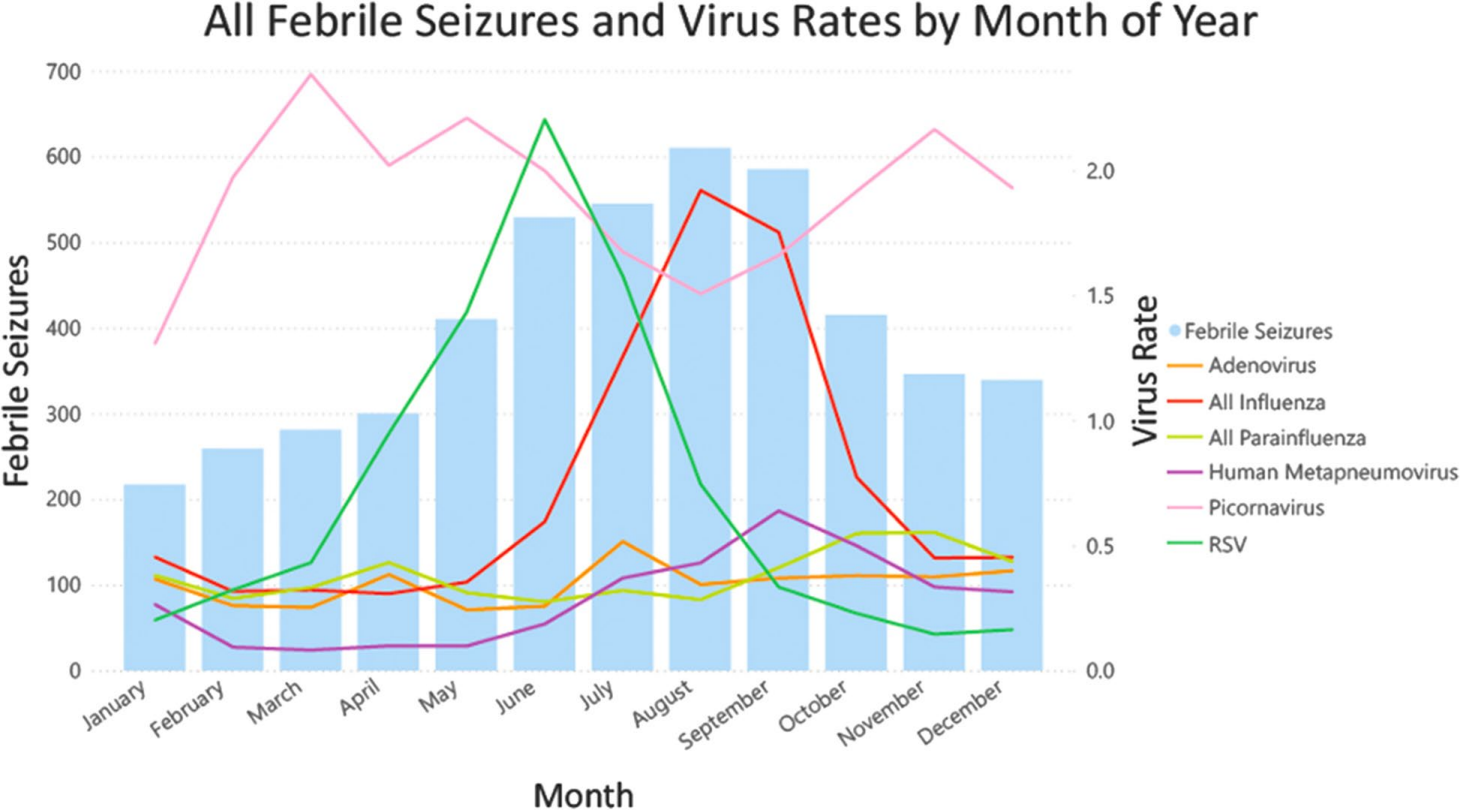
Febrile Seizure Presentations And Virus Rate 2010–2019

**Fig. 3:**
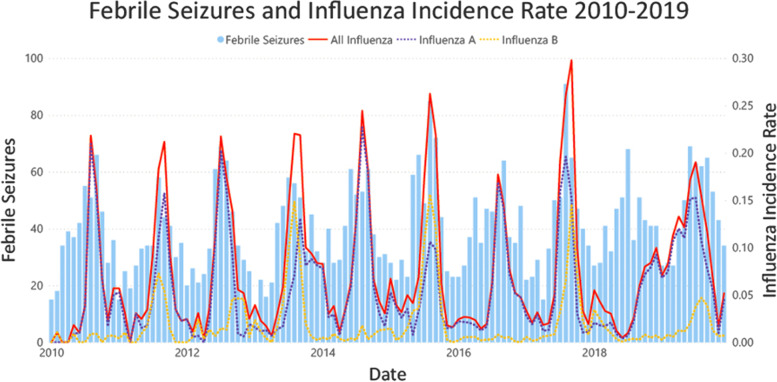
Infleunza rate and febrile seizures 2010–2019

**Fig. 4:**
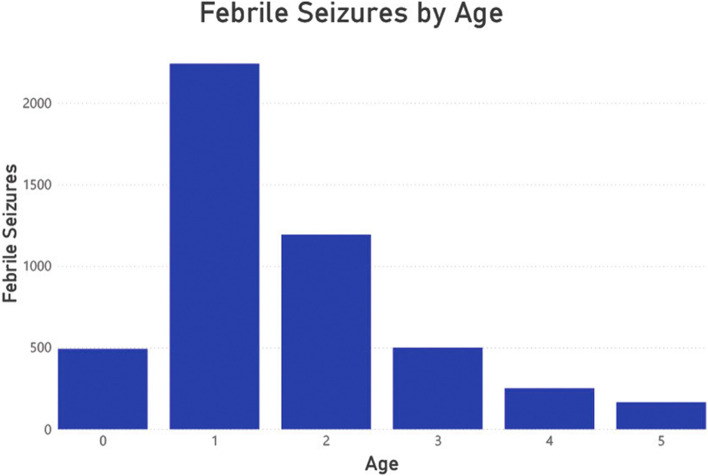
Number of febrile seizure presentations in each age group

This is the first ecologic study assessing febrile seizures with reference to contemporaneously collected multiplex viral respiratory samples from the same catchment population. We found that there was an increase in febrile seizures from May to October of each year compared to the risk of febrile seizure incidence in April, with the greatest risk in August. In Victoria, with a temperate climate, this correlates with late fall and winter seasons. These findings have been previously described and likely relate to the increased viral activity in these months, particularly Influenza, RSV and hMPV (Figs. 2 and 3) [10, 11, 13, 14]. This hypothesis is supported by the decreased risk observed in January, when fewer respiratory viruses are in circulation [34]. Notably, febrile seizures in children less than 1 year old were not significantly associated with any month of the year. One potential explanation for the absence of a trend in this age group is that febrile seizures occurring in this age group may be caused by non-seasonal viruses, such as HHV-6 [22].

There were four viruses in our model which were found to be positively associated with febrile seizure presentations. These were human metapneumovirus, influenza A and B, and RSV. Human metapneumovirus was positively associated with febrile seizures, with a RR of 1.19 (99% CI 1.07–1.33) in peak seasons, a strong and novel association. Human metapneumovirus infection has not been commonly described in association with febrile seizures and makes up a relatively small proportion of viral identifications in febrile seizure cohorts [10, 11]. However, hMPV has been associated with CNS illnesses ranging from seizures to encephalitis [35]. Further research is required to ascertain if a causal relationship exists between hMPV and febrile seizures, potentially focusing on the age distribution of children experiencing febrile seizures associated with hMPV infection.

Influenza viruses are recognised as an important cause of febrile seizures [19, 23, 29]. Our study has confirmed this with a 48% increase in febrile seizure incidence during peak Influenza A seasons and a 31% increase during peak Influenza B seasons, supporting previous findings that Influenza A is more strongly associated with febrile seizures [14, 29]. The strength of association between influenza and febrile seizures fluctuated in analysis of different subgroups. Influenza B was associated with a 1.37RR (99% CI 1.08–1.73) for severe febrile seizures, stronger than its association with mild febrile seizure occurrence. In general, Influenza viruses are associated with a greater proportion of complex febrile seizure presentations, though few studies have differentiated between Influenza A and B with regard to febrile seizure severity [10]. Nevertheless, given the small febrile seizure sample size in the subgroup analyses, the clinical significance of this association cannot be determined.

RSV had a distinct peak in May–July of each year, coinciding with the beginning of peak febrile seizure season. RSV is the most common cause of lower respiratory tract infections in children < 5 years old [36]. Moreover, it accounts for a significant proportion of respiratory viruses detected in children with febrile seizures [10]. RSV had a significant association with febrile seizures, with a 52% increase in risk in its peak seasons. This increase was consistent with previous findings [11, 25].

Ultimately, the goal of our study is to create a framework that depicts accurate risk of community health outcomes based on virus circulation. While the risk ratios of febrile seizures attributable to influenza viruses were approximately consistent with existing findings [20, 23], our risk ratios for RSV and hMPV were higher than those found in cohort studies of viral infections in febrile seizures [10, 11, 25]. One potential explanation for this is the abnormal influenza season observed in 2018, which had a delayed peak in October 2018–January 2019. Despite this, the peak of febrile seizures in winter 2018 was consistent with that of previous years. Our model likely attributed this to RSV and hMPV circulation which similarly maintained their trend of a winter peak. Thus, our findings highlight that there are multiple key viruses which may play a role in the occurrence of febrile seizures.

To our knowledge, this is the largest ecologic study relating febrile seizure incidence to circulating community viruses over such an extensive timeframe. The breadth of our inclusion criteria provided a sizeable dataset of both febrile seizure presentations and respiratory results. Respiratory PCR results were not restricted by age as the purpose was to estimate the local circulating community virus epidemiology for children presenting with febrile seizures. The 10-year time frame permitted sufficient data to confirm true seasonal patterns between viruses and febrile seizure presentations.

Additionally, the widespread use of multiplex PCR allowed collection of a large volume of concurrent sensitive data about numerous viruses [37, 38]. The interplay between the circulation of multiple viruses allowed realistic models for predicting febrile seizure incidence and the proportion attributable to specific viruses. We also determined our significance level at a low *p*-value (*p* <  0.01) to increase certainty in our findings.

The key limitation of the ecologic study design is that all associations are indirect links of febrile seizures incidence with key viruses. That is, although Influenza A and B, hMPV, and RSV circulation in the community may correlate temporally with febrile seizure presentations, our study cannot confirm that these are the causative agents associated with febrile illnesses leading to febrile seizures. In most febrile seizures presenting to emergency departments a respiratory PCR is rarely performed, and the assessment of the child usually only involves a clinical assessment to determine the cause of the seizure and rule out more sinister aetiologies [39].

**Fig. 5:**
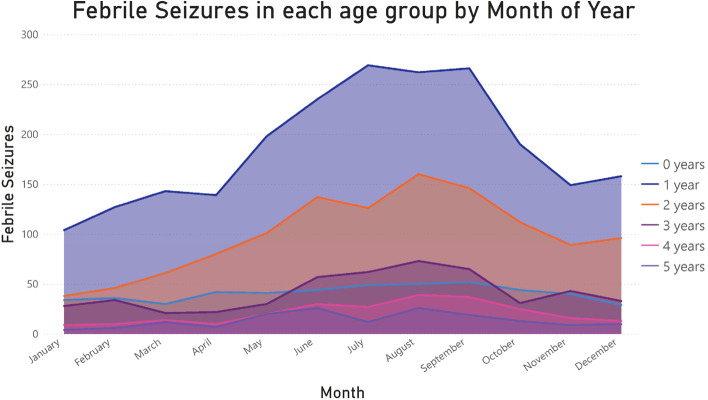
Febrile seizure seasonal trends vary in each age group

The multiplex respiratory PCR performed at Monash Health sites had limitations. This PCR test does not detect HHV-6, a virus historically associated with febrile seizures. This may have been an explanatory variable for febrile seizure presentations in children less than 1 year old, which did not demonstrate a seasonal trend (Fig. 5). Additionally, our PCR assay did not differentiate between picornaviruses (i.e. enteroviruses and rhinoviruses). This made it difficult to determine the association of enterovirus and rhinovirus circulation with febrile seizures, which have been reported in previous studies [10, 11], and may have confounded our findings by leading to overestimation of the relationship between febrile seizures and the other viruses included in our model.

There was as gradual increase in the number of PCR tests performed throughout each year of our study due to the changes in the clinical use of PCR testing. Therefore, we calculated positive virus results as a proportion of the total number of PCR tests performed each month to ensure the values remained relatively consistent throughout the 10-year period. While this may have masked some seasonal variation in virus circulation, this was the simplest way to create uniformity in our virus variables for the sake of analysis.

It is also important to consider that our risk ratio values were obtained based on a comparison of peak viral circulation and median viral circulation. While this allows the findings of our study to be contextualised for clinicians and public health physicians, it is difficult to relate these values to previous cohort studies. Thus, our model requires further adjustments to increase the interpretability of our findings, and future research will focus on different definitions of peak viral seasons.

Although Monash Health is the major health network providing care to children from southern and eastern Melbourne, it does not capture all emergency presentations or PCR tests from that region. As such, our datasets represented a subset from that region.

Our study has established a method for studying temporal relationships of viruses to an illness of interest using independent datasets. This methodology has implications for future studies of illnesses with undefined viral pathogenesis. The ability to estimate “attributable proportion” for specific viruses for conditions such as febrile seizures, offers the potential to inform organism specific disease burden and resultant health technology assessments for viral vaccines and therapeutics. These studies can inform healthcare practices and resource allocation and increase public awareness of the implications of specific viral infections, as has been done in Utah with GermWatch [40].

## Conclusion

Febrile seizures are a common and clinically important illness in young children, informing future febrile and afebrile seizure risk. Our ecologic study used modern molecular viral detection technology to analyse febrile seizure temporal patterns and relationships with viruses over a 10-year period. We found that the incidence of febrile seizures doubled in winter, which was correlated with known seasonal variation in viral activity. In addition to a novel association between febrile seizures and hMPV, Influenza A, Influenza B and RSV were associated with increased febrile seizure presentations. Adenovirus, parainfluenza viruses and picornaviruses were not associated.

Our study demonstrates that we have created a model for using population-level data to relate virus circulation to health outcomes of interest. This may be particularly useful in the future for resource allocation and to assess disease-specific health outcomes, especially given the rise of novel viruses and their implications.

### Supplementary Information

**Additional file 1.**

## Data Availability

Data sharing not applicable to this article as no datasets were generated or analysed during the current study.

## Appendix A

### Ethics

#### Study protocol

Please see Supplementary File in submission titled “Snotwatch Protocol Version 2.0”.

### Appendix C Number of respiratory PCR tests performed each year

**Table 3:** Number of each virus positive PCR and the total tests 2010–2019

Virus	2010	2011	2012	2013	2014	2015	2016	2017	2018	2019
Adenovirus	232	217	154	184	253	255	283	294	347	436
hMPV	133	164	156	138	239	303	309	354	480	586
Influenza A	195	251	361	260	712	485	737	1177	524	1904
Influenza B	14	105	96	222	58	575	44	635	115	400
Picornavirus	878	1014	1031	877	1491	1464	1539	1726	2295	3137
Parainfluenza 1	30	7	45	14	94	21	107	47	156	32
Parainfluenza 2	11	27	14	29	5	53	17	51	19	98
Parainfluenza 3	119	161	139	137	145	273	230	332	349	542
RSV	442	393	439	376	548	577	686	856	888	1008
All Viruses	2054	2339	2435	2237	3545	4006	3952	5472	5173	8143
Total Tests Performed	2960	4168	4700	4615	7920	9426	11,475	14,617	14,099	19,893

### Appendix E Subgroup poisson models

#### Analysis of mild febrile seizures

**Table 4:** RisK of mild febrile seizures by month of year

Month	Risk Ratio	99% CI	*P*-value
January	0.72	0.56, 0.93	0.001
February	0.80	0.63, 1.03	0.023
March	0.89	0.70, 1.13	0.21
April (Reference)	1.00	–	–
May^*^	1.33	1.07, 1.65	0.0008
June^*^	1.67	1.36, 2.06	< 0.0001
July*	1.76	1.44, 2.17	< 0.0001
August^*^	1.92	1.57, 2.35	< 0.0001
September^*^	1.85	1.51, 2.26	< 0.0001
October^*^	1.27	1.02, 1.58	0.0053
November	1.09	0.86, 1.36	0.35
December	1.05	0.83, 1.32	0.59

Intercept denotes estimated number of mild febrile seizures in April. Statistically significant months are denoted with *. The baseline monthly number of febrile seizures for this subgroup was 24.4

**Table 5:** Risk of mild febrile seizure presentations associated with respiratory viruses

Virus	95% vs 50% Incidence Risk	99% CI	*P*-value
Adenovirus	1.03	0.95, 1.13	0.36
hMPV^*^	1.18	1.04, 1.34	0.00091
Influenza A^*^	1.50	1.32, 1.72	< 0.0001
Influenza B^*^	1.30	1.15, 1.47	< 0.0001
Parainfluenza 1	0.94	0.77, 1.14	0.41
Parainfluenza 2	0.99	0.88, 1.11	0.83
Parainfluenza 3	1.04	0.90, 1.20	0.47
Picornavirus	1.07	0.92, 1.23	0.26
RSV^*^	1.53	1.36, 1.73	< 0.0001

Intercept denotes estimated number of mild febrile seizures per month. Statistically significant viruses are denoted with^*^. The baseline monthly number of febrile seizures for this subgroup was 15.5

#### Analysis of severe febrile seizures

**Table 6:** Risk of severe febrile seizures by month of year

Month	Risk Ratio	99% CI	*P*-value
January	0.72	0.42, 1.24	0.12
February	1.13	0.70, 1.83	0.51
March	1.15	0.71, 1.85	0.46
April (Reference)	1.00	–	–
May	1.54	0.98, 2.41	0.014
June^*^	2.17	1.42, 3.31	< 0.0001
July^*^	2.06	1.34, 3.15	< 0.0001
August^*^	2.54	1.68, 3.34	< 0.0001
September^*^	2.43	1.60, 3.68	< 0.0001
October^*^	1.91	1.24, 2.94	0.00012
November	1.46	0.93, 2.31	0.031
December	1.50	0.95, 2.36	0.021

Intercept denotes estimated severe number of febrile seizures in april. Statistically significant months are denoted with^*^. The baseline monthly number of febrile seizures for this subgroup was 5.2

**Table 7:** Risk of severe febrile seizure presentations associated with respiratory viruses

Virus	95% vs 50% Incidence Risk	99% CI	*P*-value
Adenovirus	0.93	0.80, 1.09	0.24
hMPV	1.22	0.98, 1.453	0.020
Influenza A^*^	**1.47**	1.16, 1.86	< 0.0001
Influenza B^*^	**1.37**	1.08, 1.73	< 0.0001
Parainfluenza 1	**0.93**	0.64, 1.35	0.62
Parainfluenza 2^*^	0.79	0.63, 1.00	0.0095
Parainfluenza 3	1.12	0.88, 1.42	0.22
Picornavirus	1.04	0.81, 1.33	0.68
RSV^*^	**1.55**	1.25, 1.94	< 0.0001

Intercept denotes estimated number of severe febrile seizures per month. Statistically significant viruses are denoted with^*^. The baseline monthly number of febrile seizures for this subgroup was 4.7

#### Analysis of febrile seizures in children less than one year old

**Table 8:** Risk of febrile seizures in children < 1year old by month of year

Month	Risk Ratio	99% CI	*P*-value
January	0.85	0.46, 1.56	0.48
February	0.92	0.51, 1.67	0.73
March	0.74	0.40, 1.40	0.23
April (Reference)	1.00	–	–
May	1.05	0.59, 1.87	0.82
June	1.13	0.64, 1.99	0.58
July	1.26	0.72, 2.18	0.29
August	1.26	0.72, 2.18	0.29
September	1.31	0.75, 2.26	0.21
October	1.10	0.62, 1.95	0.66
November	1.03	0.57, 1.83	0.91
December	0.72	0.38, 1.36	0.18

Intercept denotes estimated number of febrile seizures in this age group in April. The baseline monthly number of febrile seizures for this subgroup was 4.28

**Table 9:** Risk of febrile seizures in children < 1 year old associated with respiratory viruses

Virus	95% vs 50% Incidence Risk	99% CI	*P*-value
Adenovirus	1.03	0.81, 1.30	0.79
hMPV	1.16	0.82, 1.64	0.27
Influenza A	1.13	0.81, 1.58	0.35
Influenza B	1.24	0.93, 1.67	0.054
Parainfluenza 1	1.15	0.73, 1.81	0.43
Parainfluenza 2	1.04	0.77, 1.40	0.73
Parainfluenza 3	0.89	0.61, 1.30	0.42
Picornavirus	0.84	0.64, 1.10	0.096
RSV	1.07	0.76, 1.51	0.63

Intercept denotes the estimated number of febrile seizures in this age group per month. The baseline monthly number of febrile seizures for this subgroup was 4.3

#### Analysis of febrile seizures in children 1–2 years old

**Table 10:** Risk of febrile seizures in children 1–2 years old by month of year

Month	Risk Ratio	99% CI	*P*-value
January^*^	0.65	0.49, 0.86	< 0.0001
February	0.78	0.60, 1.02	0.17
March	0.92	0.71, 1.18	0.38
April (Reference)	1.00	–	–
May^*^	1.33	1.05, 1.67	0.0018
June^*^	1.68	1.34, 2.10	< 0.0001
July^*^	1.80	1.44, 2.24	< 0.0001
August^*^	1.91	1.54, 2.37	< 0.0001
September^*^	1.85	1.49, 2.30	< 0.0001
October^*^	1.37	1.09, 1.73	0.00042
November	1.06	0.83, 1.35	0.54
December	1.16	0.92, 1.48	0.10

Intercept denotes estimated number of febrile seizures in this age group in April. Statistically significant months are denoted with^*^. The baseline monthly number of febrile seizures for this subgroup was 21.9

**Table 11:** Risk of febrile seizures in children 1–2 years old associated with respiratory viruses

Virus	75% vs 25% Incidence Risk	99% CI	*P*-value
Adenovirus	1.01	0.92, 1.10	0.83
hMPV^*^	1.18	1.03, 1.35	0.0015
Influenza A^*^	1.52	1.32, 1.75	< 0.0001
Influenza B^*^	1.25	1.09, 1.42	< 0.0001
Parainfluenza 1	0.95	0.77, 1.16	0.49
Parainfluenza 2	0.91	0.81, 1.04	0.067
Parainfluenza 3	1.08	0.94, 1.25	0.16
Picornavirus	1.04	0.89, 1.21	0.56
RSV^*^	1.58	1.39, 1.79	< 0.0001

Intercept denotes the estimated number of febrile seizures in this age group per month. Statistically significant viruses are denoted with^*^. The baseline monthly number of febrile seizures for this subgroup was 14.5

#### Analysis of febrile seizures in children 3–5 years old

**Table 12:** Risk of febrile seizures in children 3–5 years old by month of year

Month	Risk Ratio	99% CI	*P*-value
January	1.03	0.57, 1.83	0.91
February	1.28	0.74, 2.22	0.24
March	1.18	0.67, 2.07	0.45
April (Reference)	1.00	–	–
May^*^	1.77	1.06, 2.96	< 0.0044
June^*^	2.87	1.78, 4.64	< 0.0001
July^*^	2.56	1.58, 4.17	< 0.0001
August^*^	3.49	2.18, 5.57	< 0.0001
September^*^	3.00	1.86, 4.83	< 0.0001
October^*^	1.74	1.04, 2.93	0.0056
November^*^	1.74	1.04, 2.93	0.0056
December	1.44	0.84, 2.46	0.083

Intercept denotes estimated number of febrile seizures in this age group in April. Statistically significant months are denoted with^*^. The baseline monthly number of febrile seizures for this subgroup was 3.9

**Table 13:** Risk of febrile seizures in children 3–5 years old associated with respiratory viruses

Virus	95% vs 50% Incidence Risk	99% CI	*P*-value
Adenovirus	0.83	0.67, 1.04	0.03
hMPV	1.17	0.91, 1.51	0.0990
Influenza A^*^	1.54	1.20, 1.98	< 0.0001
Influenza B^*^	1.44	1.13, 1.84	< 0.0001
Parainfluenza 1	0.78	0.51, 1.19	0.13
Parainfluenza 2	0.94	0.74, 1.20	0.52
Parainfluenza 3	1.13	0.86, 1.49	0.26
Picornavirus	0.88	0.69, 1.14	0.21
RSV^*^	1.67	1.31, 2.12	< 0.0001

Intercept denotes the estimated number of febrile seizures in this age group per month. Statistically significant viruses are denoted with^*^. The baseline monthly number of febrile seizures for this subgroup was 5.3

## Abbreviations

CI: Confidence Interval
CNS: Central Nervous System
ED: Emergency Department
ERM: Ethical Review Manager
HHV-6: Human Herpesvirus-6
hMPV: Human Metapneumovirus
HREC: Human Research Ethics Committee
ICD-10 AM: International Statistical Classification of Diseases and Related Health Problems, Tenth Revision, Australian Modification
MOY: Month of Year
NMA: National Mutual Acceptance
PCR: Polymerase Chain Reaction
RR: Risk Ratio
RSV: Respiratory Syncytial Virus
URN: Unit Record Number
URTIs: Upper Respiratory Tract Infections
