# Supplementary material for: Snotwatch: an ecological analysis of the relationship between febrile seizures and respiratory virus activity
Source: BMC Pediatr. 2022 Jun 22;22:359. doi: 10.1186/s12887-022-03222-4 (PMC9215000; doi:10.1186/s12887-022-03222-4)
Supplement: Supplementary file 1 — Additional file 1. [file 12887_2022_3222_MOESM1_ESM.docx]

**STUDY PROTOCOL**

**SNOTWATCH:**

**Mapping real-time respiratory microbiology information to inform health outcomes**

| ERM 53611  Monash Health Ethics Reference RES-19-0000333L-53611  Version 2.0  Date: 18 October 2021  **Document history:**   \| **Version Number and Date** \| **Summary of Changes** \| \| --- \| --- \| \| **Version 1.0** 23/05/2018 \| Document created by original research team \| \| **Version 2.0** 18/11/2021 \| - Document created by investigators using new protocol template - Addition of information under Study Oversight; Consent; Governance and Quality Assurance measures; Statistical Methods (Sample Size) - Addition of Data and Information Management measures including Study Schema - Addition of an Appendix detailing source data variables \| |
| --- | --- | --- | --- | --- | --- | --- |
| \| Principal Investigator: \| Prof Jim Buttery  Centre for Health Analytics, Murdoch Children’s Research Institute \| \| --- \| --- \| \| Associate Investigators: \| SNOTWATCH Asthma:  Allen Cheng, Danielle Mazza, Anton Peleg, Harvey Newnham, Grant Russell, Adelaide Grenfell  Collaborating laboratories and co-investigators:  Monash Health (Tony Korman), Alfred Health (Adam Jenney), Royal Children’s Hospital (RCH – Andrew Daley), Barwon Health (Eugene Athan), Eastern Health (Roy Chean), Melbourne Health (Katherine Bond), Victorian Infectious Diseases Reference Laboratory (VIDRL)  Collaborating data partners and investigators:  Department of Health and Human Services (DHHS – Jim Black, Brett Sutton, Lucinda Franklin), POLAR GP (Chris Pearce), Monash Health (Adam West), Alfred Health (Harvey Newnham), RCH (Franz Babl), Ambulance Victoria (Karen Smith), Health Direct (no named investigator). \| \| Funding: \| The Royal Children’s Hospital Foundation \| \| Study coordinating site: \| Murdoch Children’s Research Institute (MCRI), Centre for Health Analytics, Royal Children’s Hospital (RCH), 50 Flemington Rd Parkville 3052 \| \| Ethics and governance site: \| Monash Centre for Health Research and Implementation (MCHRI), Monash University, Monash Health 246 Clayton Road, Clayton VIC 3168 \| \| Research Team \| Rana Sawires, PhD Student Investigator  Dr Josh Osowicki, Paediatric Infectious Disease Physician  Deniz Akin, Research Assistant \|   **CONFIDENTIAL**  This document is confidential and is the property of Murdoch Children’s Research Institute. No part of it may be transmitted, reproduced, published, or used without prior written authorisation from the institution.  **Statement of Compliance**  This study will be conducted in compliance with all stipulation of this protocol, the conditions of the ethics committee approval, the NHMRC National Statement on Ethical Conduct in Human Research (2007 and all updates), applicable national and local regulations and in the spirit of the Integrated Addendum to ICH E6 (R1): Guideline for Good Clinical Practice E6 (R2), dated 9 November 2016 annotated with TGA comments. |

TABLE OF Contents

[PROTOCOL SYNOPSIS 6](#_Toc88819207)

[GLOSSARY OF ABBREVIATIONS 7](#_Toc88819208)

[INVESTIGATOR AGREEMENT 8](#_Toc88819209)

[1. ADMINISTRATIVE INFORMATION 9](#_Toc88819210)

[1.1. Registration of observational research 9](#_Toc88819211)

[1.2. Expected duration of study 9](#_Toc88819212)

[2. INTRODUCTION AND BACKGROUND 9](#_Toc88819213)

[2.1. Background and rationale 9](#_Toc88819214)

[3. STUDY OBJECTIVES AND OUTCOMES 10](#_Toc88819215)

[3.1. Objectives 10](#_Toc88819216)

[3.1.1. Primary objective 10](#_Toc88819217)

[3.1.2. Secondary objectives 10](#_Toc88819218)

[4. STUDY DESIGN AND METHODOLOGY 10](#_Toc88819219)

[4.1. Study design schema 10](#_Toc88819220)

[4.2. Study Schema 10](#_Toc88819221)

[4.3. Retrospective Study Design 11](#_Toc88819222)

[4.3.1. Microbiology extraction and de-identification 11](#_Toc88819223)

[4.3.2. Microbiology results secure uploading 11](#_Toc88819224)

[4.3.3. Clinical presentation data extraction and de-identification 11](#_Toc88819225)

[4.4. Prospective Study Design 13](#_Toc88819226)

[4.4.1. Microbiology extraction and de-identification 13](#_Toc88819227)

[4.4.2. Microbiology results secure uploading 13](#_Toc88819228)

[4.4.1.2. Clinical presentation data extraction and de-identification 14](#_Toc88819229)

[4.4.3. Data merging and cleaning 16](#_Toc88819230)

[4.5. Study population 16](#_Toc88819231)

[4.5.6. Sample Size 16](#_Toc88819232)

[4.5.7. Inclusion Criteria 16](#_Toc88819233)

[4.5.8. Exclusion Criteria 16](#_Toc88819234)

[4.6. STUDY VISITS AND PROCEDURES 17](#_Toc88819235)

[4.6.1. Schedule of data collection 17](#_Toc88819236)

[5. Participant withdrawals and losses to follow up 17](#_Toc88819237)

[5.1. Withdrawal of consent 17](#_Toc88819238)

[5.2. Losses to follow-up 17](#_Toc88819239)

[5.3. Replacements 17](#_Toc88819240)

[5.4. Study Closure 17](#_Toc88819241)

[6. PRIVACY AND CONFIDENTIALITY 18](#_Toc88819242)

[6.1. Consent 18](#_Toc88819243)

[6.2. Informed Consent Process 19](#_Toc88819244)

[7. POTENTIAL RISKS RELATED TO STUDY CONDUCT 19](#_Toc88819245)

[7.1. Risk to participants 19](#_Toc88819246)

[7.2. Benefits to participants 20](#_Toc88819247)

[8. CONFLICT OF INTEREST 20](#_Toc88819248)

[9. DATA AND INFORMATION MANAGEMENT 20](#_Toc88819249)

[9.1. Publication and Presentation 20](#_Toc88819250)

[9.2. Overview 20](#_Toc88819251)

[9.2.1. Data management 20](#_Toc88819252)

[Data confidentiality 22](#_Toc88819253)

[10. STUDY OVERSIGHT 23](#_Toc88819254)

[10.1. Governance structure 23](#_Toc88819255)

[10.2. Quality management, assurance and control 23](#_Toc88819256)

[11. DATA ANALYSIS 23](#_Toc88819257)

[11.1. Sample size and statistical power 23](#_Toc88819258)

[11.2. Statistical methods 24](#_Toc88819259)

[11.3. Population to be analysed 24](#_Toc88819260)

[11.3.1. Handling of missing data 24](#_Toc88819261)

[11.3.2. Data limitations 24](#_Toc88819262)

[12. ETHICS AND DISSEMINATION 25](#_Toc88819263)

[12. 1 Research Ethics Approval & Local Governance Authorisation 25](#_Toc88819264)

[12.2. Amendments to the protocol 25](#_Toc88819265)

[12.3. Protocol deviations and serious breaches 25](#_Toc88819266)

[13. DATA SUPPLIER REIMBURSEMENT 26](#_Toc88819267)

[14. FINANCIAL DISCLOSURE AND CONFLICTS OF INTEREST 26](#_Toc88819268)

[15. PUBLICATION AND INTELLECTUAL PROPERTY PLAN 26](#_Toc88819269)

[15.1. Dissemination and translation plan 26](#_Toc88819270)

[15.2. Dissemination of results to participants 26](#_Toc88819271)

[15.3. Intellectual property 27](#_Toc88819272)

[16 APPENDIX 28](#_Toc88819273)

[16.1. Requested variables from each dataset 28](#_Toc88819274)

# PROTOCOL SYNOPSIS

| **TITLE** | SNOTWATCH: Mapping real-time respiratory microbiology information to inform health outcomes |
| --- | --- |
| **STUDY DESCRIPTION** | We aim to use retrospective, unlinked datasets which report on health outcomes and respiratory PCR results. These two datasets are linked based on date of presentation as well as location to create one large dataset.  This enables easy data visualisation and statistical analysis.  Statistical analysis involves fit testing of our data and application of techniques which determine how well viruses can predict the health outcomes of interest. Statistical analysis may be conducted on temporal data only or on spatiotemporal data. |
| **OBJECTIVES** | To examine and describe organism specific epidemiology of respiratory and gastrointestinal positive tests from all participating laboratories.  To describe the association of organisms in time and place with health service utilisation and presentations. |
| **OUTCOMES AND OUTCOME MEASURES** | - Outcomes are the statistical association between respiratory virus and a health outcome of interest. This will be stratified based on temporal-only association and spatiotemporal associations. - Outcomes also include qualitative associations found between viruses and health outcomes, represented visually. - Associations between health outcomes and month of the year may also be calculated as appropriate. - Health outcomes may also be analysed in relation to other predictive variables depending on the specific illness being studied. |
| **STUDY POPULATION** | De-identified pathology and clinical presentations data received from the entire State of Victoria |
| **DESCRIPTION OF SITES ENROLLING PARTICIPANTS** | Multiple levels of health care including state-wide infectious disease surveillance services, telephone health advice operators, private and public pathology services, general practitioners (GP), ambulance services, emergency department and hospital admission data. |
| **STUDY DURATION** | SNOTWATCH is an ongoing platform for analysing various health outcomes in the community with virus circulation. |
| **PARTICIPANT DURATION** | As per each institutional research agreement. |

# GLOSSARY OF ABBREVIATIONS

| PCR | Polymerase Chain Reaction |
| --- | --- |
| RSV | Respiratory Syncytial Virus |
| PIV | Parainfluenza Virus |
| hMPV | Human Metapneumovirus |
| Flu | Influenza |
| ED | Emergency Department |
| VAED | Victorian Admitted Episodes Dataset |
| VEMD | Victorian Emergency Minimum Dataset |
| PREDICT | Paediatric Research in Emergency Departments International Collaborative |
| RHEMMS | Rapid Health Emergency Medicine Monitoring System |
| POLAR | Population Level Analysis and Reporting Tool |
| VIDRL | Victorian Infectious Diseases Reporting Laboratory |

# INVESTIGATOR AGREEMENT

I have read the protocol entitled “*SNOTWATCH – Mapping real-time respiratory microbiology information to inform health outcomes*”.

By signing this protocol, I agree to conduct the study, after approval by a Human Research Ethics Committee or Institutional Review Board (as appropriate), in accordance with the protocol and:

- the principles of the Declaration of Helsinki
- the NHMRC National Statement on Ethical Conduct in Human Research (2007 and all updates)
- the Australian Codefor the Responsible Conduct of Research (NHMRC, 2007 and all updates)
- and in the spirit of the good clinical practice guidelines adopted by the TGA [Integrated Addendum to ICH E6 (R1): Guideline for Good Clinical Practice E6 (R2), dated 9 November 2016 annotated with TGA comments].

Changes to the protocol will only be implemented after written approval is received from the Human Research Ethics Committee or Institutional Review Board (as appropriate), with the exception of medical emergencies.

I will ensure that study staff fully understand and follow the protocol and evidence of their training is documented on the study training log.

| **Name** | **Organisation** | **Email** | **Role** | **Signature** | **Date signed** |
| --- | --- | --- | --- | --- | --- |
| Professor Jim Buttery | Murdoch Children’s Research Institute | jim.buttery@mcri.edu.au | Principal investigator | 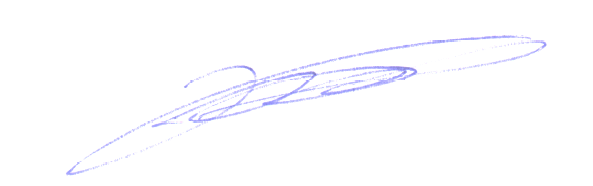 | 18/10/2021 |
| Dr Joshua Osowicki | Murdoch Children’s Research Institute | joshua.osowicki@mcri.edu.au | Paediatric Infectious Diseases Physician | 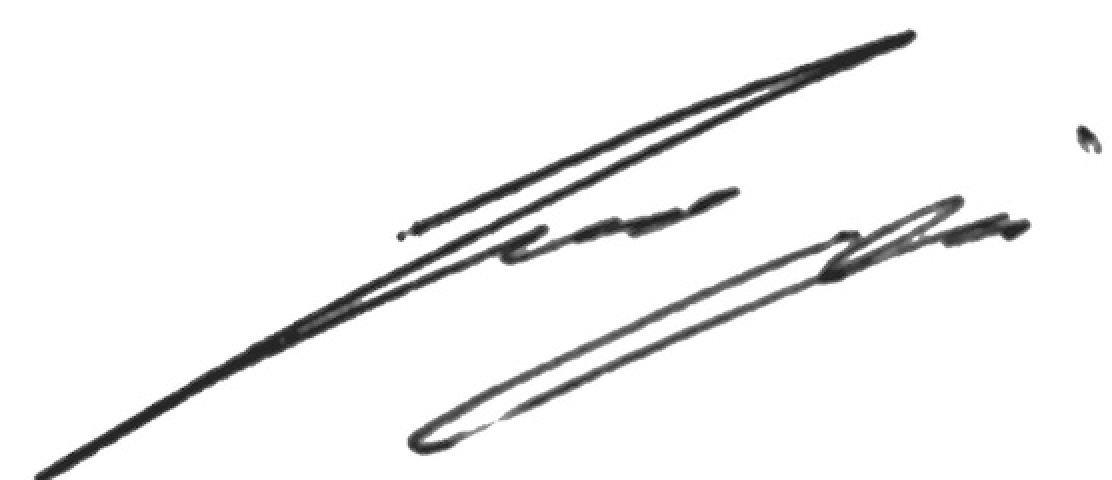 | 25/11/201 |
| Rana Sawires | Murdoch Children’s Research Institute | rana.sawires@mcri.edu.au | PhD Student | 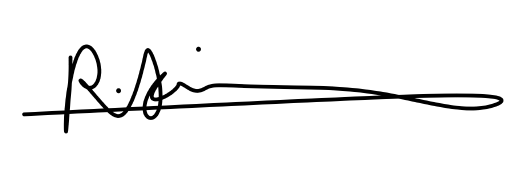 | 26/11/2021 |
| Deniz Akin | Murdoch Children’s Research Institute | deniz.akin@mcri.edu.au | Research Assistant | 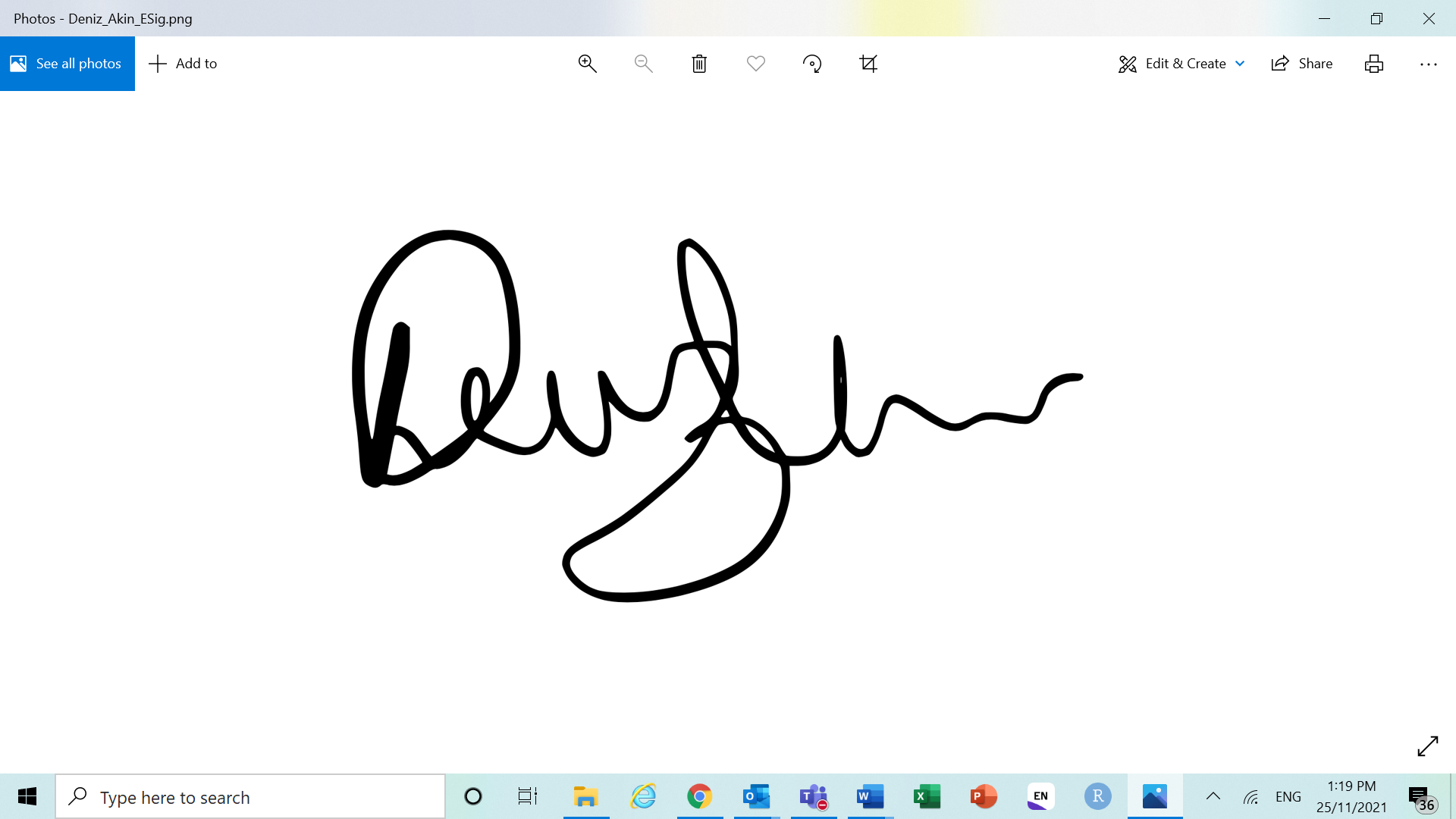 | 18/10/2021 |

# ADMINISTRATIVE INFORMATION

# Registration of observational research

On behalf of the Sponsor, MCRI, the Principal Investigator (PI) will undertake and/or oversee those Sponsor responsibilities delegated by the Sponsor. The PI will also ensure that each Investigator at the participating sites conducts the study in compliance with the protocol, relevant approvals and regulatory requirements.

| **Study Sponsor** | MCRI |
| --- | --- |
| **Contact name** | Prof Jim Buttery |
| **Address** | Royal Children’s Hospital, 50 Flemington Road, Parkville VIC 3052 |

# Expected duration of study

The SNOTWATCH project will be an ongoing epidemiological analysis with no end date. Viability of the project will be assessed every four years.

Since the study initiation in 2019 and on-going enrolment of data partners, retrospective data will be sought from each participating Site/Data Partner dating back 7-10 years from the date of their enrolment into the SNOTWATCH project. Enrolment of sites for prospective data-linkage will remain open until the SNOTWATCH aims are met. The duration of this study for each participating site will be ongoing or as determined as per the research agreements. The PI will ensure that all HRECs and RGOs as well as regulatory and funding bodies have been notified at the closure of the SNOTWATCH project.

*See – Section 5. for Participant Withdrawal.*

# INTRODUCTION AND BACKGROUND

# Background and rationale

Recent years have seen a widespread uptake of respiratory (and gastro-intestinal) pathogen molecular diagnostic tests that test for multiple potential pathogens for each sample. Not only does this increase the breadth or organisms tested for, but the increased sensitivity of this testing for DNA or RNA of these organisms has vastly increased the number of positive tests.

This has allowed the availability of surrogate population level data regarding the distribution of many common pathogens across time and place, with most hospitals and community pathogen providers in Victoria utilising these assays. It is now feasible technically to de-identify these data and amalgamate all results to improve our understanding of how these organisms circulate each year.

At the same time, our ability to examine location specific health events from multiple levels of health care provision has improved. These include calls to telephone health advice lines, general practitioner (GP) presentations, ambulance encounters, emergency department presentations and hospital admission data.

This now means we have the opportunity to examine associations between potential pathogens and patterns of health care presentations using geo-temporal statistical methodologies with completely deidentified, non-linked data. This has the advantage of observing all patients’ privacy while still having the potential to upload, aggregate and examine real-time data.

1. STUDY OBJECTIVES AND OUTCOMES

# Objectives

The objectives of this study are:

To examine and describe organism specific epidemiology of respiratory and gastrointestinal positive tests from all participating laboratories.

To describe the association of organisms in time and place with health service utilisation and presentations.

# Primary objective

To describe the retrospective and ongoing prospective geo-temporal organism and age specific epidemiology of de-identified respiratory and gastrointestinal multiplex polymerase chain reaction (PCR) positive results from all participating laboratories across Victoria.

# Secondary objectives

The secondary objectives of this study are:

To use retrospective data, to evaluate the geo-temporal association of organism specific activity with health outcomes and health service utilisation from multiple levels of healthcare, including hospital admission and emergency presentations, ambulance service provision, GP presentations and telephone health advice requests.

To publish the organism specific epidemiology for continuously updated open access, for example on a website accompanied with related health information for community members and primary care practitioners.

# 4. STUDY DESIGN AND METHODOLOGY

# 4.1. Study design schema

This study will be conducted in two phases. First, the retrospective phase will involve collection, synthesis, and analysis of retrospective data to establish appropriate statistical analysis methodologies and potential approach to prospective data. Following establishment of this methodology, the prospective phase will begin, applying the modelling techniques developed in the retrospective phase.

# 4.2. Study Schema


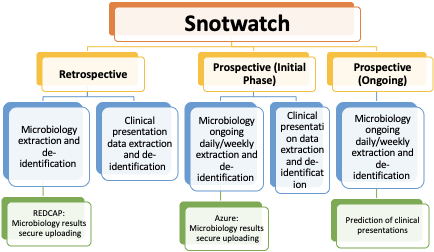


# 4.3. Retrospective Study Design

### 4.3.1. Microbiology extraction and de-identification

Retrospective results for multiplex PCR assays and other upper airway specimens (e.g., throat swab culture) will be extracted from each pathology system within each organisation’s firewall, then de-identified, with further potentially identifiable information being aggregated (e.g., birthdates calculated into closest year of age).

### 4.3.2. Microbiology results secure uploading

De-identifiable aggregated fields for each pathology tests will be uploaded into a secure REDCap

database with multi-factor authentication. The uploaded fields will be:

- Age in years (or less than one year of age: <6m, 6m-11m)
- Postcode of residence
- Gender
- Date of test
- All positive results for that test (e.g., RSV and influenza positive)

### 4.3.3. Clinical presentation data extraction and de-identification

The following table delineates the intended datasets for inclusion into the SNOTWATCH project. Refer to Appendix 16.1. for the requested variables for each dataset.

| Victorian Hospital Admission Data | Data will be obtained from the Victorian Admitted Episode Dataset (VAED) regarding acute admissions for conditions potentially related to respiratory infections or gastrointestinal infections, including (but not limited to) pneumonia, exacerbations of chronic obstructive pulmonary disease, asthma, bronchiolitis, gastroenteritis, fever, febrile convulsions, encephalitis, sepsis, invasive Group A streptococcal disease and acute myocardial episodes. As other opportunities arise, arrangements will be made to bring additional data sources into the SNOTWATCH initiative, depending on their relevance to specific conditions e.g., BloodSTAR for intravenous immunoglobulin usage for Kawasaki disease). |
| --- | --- |
| Victorian Emergency Department Presentation Data | Data will be obtained from a variety of emergency department data sources.  Retrospective data will be obtained from the Victorian Emergency Minimum Dataset (VEMD) regarding acute admissions for conditions potentially related to respiratory infections or gastrointestinal infections, including (but not limited to) pneumonia, exacerbations of chronic obstructive pulmonary disease, asthma, bronchiolitis, gastroenteritis, fever, febrile convulsions, encephalitis, sepsis, invasive Group A streptococcal disease and acute myocardial episodes. |
| Site-specific Emergency Department Presentation Data | ED department software will be accessed from Victorian hospitals including (but not limited to) Alfred Health (Cerner), Monash Health (Symphony), the Royal Children’s Hospital (Epic) for presentations potentially related to respiratory infections or gastrointestinal infections, including (but not limited to) pneumonia, exacerbations of chronic obstructive pulmonary disease, asthma, bronchiolitis, gastroenteritis, fever, febrile convulsions, encephalitis, sepsis, invasive Group A streptococcal disease and acute myocardial episodes. |
| Rapid Health Emergency Medicine Monitoring System (RHEMMS) | RHEMMS, currently under development at DHHS Victoria, is a near real-time emergency department system centralising information from many Victorian EDs. Originally developed to detect a further episode of thunderstorm asthma activity increase as soon as possible, limited information regarding all presentations is proposed to be de-identified, encrypted and sent to the RHEMMS server in the secure DHHS Azure cloud. |
| POLAR GP General practice presentation data | Outcome Health’s POLAR GP is a collaboration between Outcome Health and participating Primary Health Networks (PHNs) that provides quality benchmark information to each practice and PHN, as well as relevant business relevant information back to each practice. De-identified data from consented general practices within participating Primary Health Networks is extracted using the “Hummingbird” extraction system from common primary care software (e.g. Best Practice, Medical Director). This data is then stored at a secure, central data warehouse. Data stored in the data warehouse is not re-identifiable outside of the GP practice it originated from, but multiple encounters by the same individual are recorded as being by the same person. Presentation diagnosis data (if entered) is classified into SNOMED categories. |
| Tele-health Data | Telephone health advice data (HealthDirect, Nurse-on-Call) is logged from telephone calls from consumers into a categorical and free text database by trained nurses. |
| Ambulance Victoria data | De-identified data from Ambulance Victoria data will be obtained from calls to ambulance Victoria recorded by telephone triage staff and ambulance attendances recorded by ambulance officers. |
| Victorian Hospital Admission Data | Data will be obtained from the Victorian Admitted Episode Dataset (VAED) regarding acute admissions for conditions potentially related to respiratory infections or gastrointestinal infections, including (but not limited to) pneumonia, exacerbations of chronic obstructive pulmonary disease, asthma, bronchiolitis, gastroenteritis, fever, febrile convulsions, encephalitis, sepsis, invasive Group A streptococcal disease and acute myocardial episodes. As other opportunities arise, arrangements will be made to bring additional data sources into the SNOTWATCH initiative, depending on their relevance to specific conditions e.g., BloodSTAR for intravenous immunoglobulin usage for Kawasaki disease). |
| Victorian Emergency Department Presentation Data | Data will be obtained from a variety of emergency department data sources.  Retrospective data will be obtained from the Victorian Emergency Minimum Dataset (VEMD) regarding acute admissions for conditions potentially related to respiratory infections or gastrointestinal infections, including (but not limited to) pneumonia, exacerbations of chronic obstructive pulmonary disease, asthma, bronchiolitis, gastroenteritis, fever, febrile convulsions, encephalitis, sepsis, invasive Group A streptococcal disease and acute myocardial episodes. |
| Site-specific Emergency Department Presentation Data | ED department software will be accessed from Victorian hospitals including (but not limited to) Alfred Health (Cerner), Monash Health (Symphony), the Royal Children’s Hospital (Epic) for presentations potentially related to respiratory infections or gastrointestinal infections, including (but not limited to) pneumonia, exacerbations of chronic obstructive pulmonary disease, asthma, bronchiolitis, gastroenteritis, fever, febrile convulsions, encephalitis, sepsis, invasive Group A streptococcal disease and acute myocardial episodes. |
| Rapid Health Emergency Medicine Monitoring System (RHEMMS) | RHEMMS, currently under development at DHHS Victoria, is a near real-time emergency department system centralising information from many Victorian EDs. Originally developed to detect a further episode of thunderstorm asthma activity increase as soon as possible, limited information regarding all presentations is proposed to be de-identified, encrypted and sent to the RHEMMS server in the secure DHHS Azure cloud. |
| POLAR GP General practice presentation data | Outcome Health’s POLAR GP is a collaboration between Outcome Health and participating Primary Health Networks (PHNs) that provides quality benchmark information to each practice and PHN, as well as relevant business relevant information back to each practice. De-identified data from consented general practices within participating Primary Health Networks is extracted using the “Hummingbird” extraction system from common primary care software (e.g. Best Practice, Medical Director). This data is then stored at a secure, central data warehouse. Data stored in the data warehouse is not re-identifiable outside of the GP practice it originated from, but multiple encounters by the same individual are recorded as being by the same person. Presentation diagnosis data (if entered) is classified into SNOMED categories. |
| Tele-health Data | Telephone health advice data (HealthDirect, Nurse-on-Call) is logged from telephone calls from consumers into a categorical and free text database by trained nurses. |
| Ambulance Victoria data | De-identified data from Ambulance Victoria data will be obtained from calls to ambulance Victoria recorded by telephone triage staff and ambulance attendances recorded by ambulance officers. |

## 4.4. Prospective Study Design

### 4.4.1. Microbiology extraction and de-identification

Daily prospective results for multiplex PCR assays and other upper airway specimens (e.g., throat swab culture) will be extracted from each pathology system within each organisation’s firewall, then de-identified, with further potentially identifiable information being aggregated (e.g., birthdates calculated into closest year of age).

# 4.4.2. Microbiology results secure uploading

De-identifiable aggregated fields for each pathology test will be uploaded into a secure server within the Victorian DHHS Azure secure cloud system daily. The uploaded fields will be:

- Age in years (or if less than one year of age: <6m, 6m-11m)
- Postcode of residence
- Gender
- Date of test

All positive results for that test (e.g., RSV and influenza positive)

# 4.4.1.2. Clinical presentation data extraction and de-identification

The following table delineates the intended datasets for inclusion into the SNOTWATCH project. Refer to Appendix 16.1. for the requested variables for each dataset.

| Victorian Hospital Admission Data | Data will be obtained from the Victorian Admitted Episode Dataset (VAED) regarding acute admissions for conditions potentially related to respiratory infections or gastrointestinal infections, including (but not limited to) pneumonia, exacerbations of chronic obstructive pulmonary disease, asthma, bronchiolitis, gastroenteritis, fever, febrile convulsions, encephalitis, sepsis, invasive Group A streptococcal disease and acute myocardial episodes. Other de-identified data fields will also be included as opportunities arise and arrangements will be made to bring additional data sources into the SNOTWATCH initiative, depending on their relevance to specific conditions e.g., BloodSTAR for intravenous immunoglobulin usage for Kawasaki disease). |
| --- | --- |
| Victorian Emergency Department Presentation Data | Data will be obtained from a variety of emergency department data sources.  Retrospective data will be obtained from the Victorian Emergency Minimum Dataset (VEMD) regarding acute admissions for conditions potentially related to respiratory infections or gastrointestinal infections, including (but not limited to) pneumonia, exacerbations of chronic obstructive pulmonary disease, asthma, bronchiolitis, gastroenteritis, fever, febrile convulsions, encephalitis, sepsis, invasive Group A streptococcal disease and acute myocardial episodes. |
| Site-specific Emergency Department Presentation Data | ED department software will be accessed from Victorian hospitals including (but not limited to) Alfred Health (Cerner), Monash Health (Symphony), the Royal Children’s Hospital (Epic) for presentations potentially related to respiratory infections or gastrointestinal infections, including (but not limited to) pneumonia, exacerbations of chronic obstructive pulmonary disease, asthma, bronchiolitis, gastroenteritis, fever, febrile convulsions, encephalitis, sepsis, invasive Group A streptococcal disease and acute myocardial episodes. |
| Rapid Health Emergency Medicine Monitoring System (RHEMMS) | RHEMMS, currently under development at DHHS Victoria, is a near real-time emergency department system centralising information from many Victorian EDs. Originally developed to detect a further episode of thunderstorm asthma activity increase as soon as possible, limited information regarding all presentations is proposed to be de-identified, encrypted and sent to the RHEMMS server in the secure DHHS Azure cloud. |
| POLAR GP General practice presentation data | Outcome Health’s POLAR GP is a collaboration between Outcome Health and participating Primary Health Networks (PHNs) that provides quality benchmark information to each practice and PHN, as well as relevant business relevant information back to each practice. De-identified data from consented general practices within participating Primary Health Networks is extracted using the “Hummingbird” extraction system from common primary care software (e.g. Best Practice, Medical Director). This data is then stored at a secure, central data warehouse. Data stored in the data warehouse is not re-identifiable outside of the GP practice it originated from, but multiple encounters by the same individual are recorded as being by the same person. Presentation diagnosis data (if entered) is classified into SNOMED categories. |
| Tele-health Data | Telephone health advice data (HealthDirect, Nurse-on-Call) is logged from telephone calls from consumers into a categorical and free text database by trained nurses. |
| Ambulance Victoria data | De-identified data from Ambulance Victoria data will be obtained from calls to ambulance Victoria recorded by telephone triage staff and ambulance attendances recorded by ambulance officers. |
| Victorian Hospital Admission Data | Data will be obtained from the Victorian Admitted Episode Dataset (VAED) regarding acute admissions for conditions potentially related to respiratory infections or gastrointestinal infections, including (but not limited to) pneumonia, exacerbations of chronic obstructive pulmonary disease, asthma, bronchiolitis, gastroenteritis, fever, febrile convulsions, encephalitis, sepsis, invasive Group A streptococcal disease and acute myocardial episodes. Other de-identified data fields will also be included as opportunities arise and arrangements will be made to bring additional data sources into the SNOTWATCH initiative, depending on their relevance to specific conditions e.g., BloodSTAR for intravenous immunoglobulin usage for Kawasaki disease). |
| Victorian Emergency Department Presentation Data | Data will be obtained from a variety of emergency department data sources.  Retrospective data will be obtained from the Victorian Emergency Minimum Dataset (VEMD) regarding acute admissions for conditions potentially related to respiratory infections or gastrointestinal infections, including (but not limited to) pneumonia, exacerbations of chronic obstructive pulmonary disease, asthma, bronchiolitis, gastroenteritis, fever, febrile convulsions, encephalitis, sepsis, invasive Group A streptococcal disease and acute myocardial episodes. |
| Site-specific Emergency Department Presentation Data | ED department software will be accessed from Victorian hospitals including (but not limited to) Alfred Health (Cerner), Monash Health (Symphony), the Royal Children’s Hospital (Epic) for presentations potentially related to respiratory infections or gastrointestinal infections, including (but not limited to) pneumonia, exacerbations of chronic obstructive pulmonary disease, asthma, bronchiolitis, gastroenteritis, fever, febrile convulsions, encephalitis, sepsis, invasive Group A streptococcal disease and acute myocardial episodes. |
| Rapid Health Emergency Medicine Monitoring System (RHEMMS) | RHEMMS, currently under development at DHHS Victoria, is a near real-time emergency department system centralising information from many Victorian EDs. Originally developed to detect a further episode of thunderstorm asthma activity increase as soon as possible, limited information regarding all presentations is proposed to be de-identified, encrypted and sent to the RHEMMS server in the secure DHHS Azure cloud. |
| POLAR GP General practice presentation data | Outcome Health’s POLAR GP is a collaboration between Outcome Health and participating Primary Health Networks (PHNs) that provides quality benchmark information to each practice and PHN, as well as relevant business relevant information back to each practice. De-identified data from consented general practices within participating Primary Health Networks is extracted using the “Hummingbird” extraction system from common primary care software (e.g. Best Practice, Medical Director). This data is then stored at a secure, central data warehouse. Data stored in the data warehouse is not re-identifiable outside of the GP practice it originated from, but multiple encounters by the same individual are recorded as being by the same person. Presentation diagnosis data (if entered) is classified into SNOMED categories. |
| Tele-health Data | Telephone health advice data (HealthDirect, Nurse-on-Call) is logged from telephone calls from consumers into a categorical and free text database by trained nurses. |
| Ambulance Victoria data | De-identified data from Ambulance Victoria data will be obtained from calls to ambulance Victoria recorded by telephone triage staff and ambulance attendances recorded by ambulance officers. |

### 4.4.3. Data merging and cleaning

Retrospective data will be combined within a Microsoft PowerBI framework to enable statistical analysis, visual presentation, and entry into associated ArcGIS software for mapping purposes. SatScan software will be used to assess geo-temporal correlation. R (coding language) will be utilised through R studio to perform further statistical analysis and compare statistical methods. Natural language processing of free text data such as ED triage presentation data will be assessed against presenting complaint notes from the 3 ED Departmental software records to improve the interpretation of triage text.

## 4.5. Study population

### 4.5.6. Sample Size

Microbiological data from participating pathology services is aimed to encompass an unknown proportion representative of the Victorian population. VAED and VEMD captures more than 95% of all (public and private) episodes of care in Victoria. Full de-identified emergency data will only be available from RCH, Monash Health and Alfred Health Networks.

### 4.5.7. Inclusion Criteria

Any respiratory or gastrointestinal multiplex PCR assay microbiological test performed by participating pathology services may be included. All VAED and VEMD records, and all ED encounters from participating datasets will be included. POLAR GP participating practices have an opt-in mechanism for declaring they are willing for their de-identified data (which does not identify the practice or GP) to be utilized for POLAR GP research council approved research projects. Individual patients at each practice have the ability to opt-out, in which case their data is not uploaded to the POLAR-GP data warehouse and will not be available for research.

### 4.5.8. Exclusion Criteria

Patients who opt out of POLAR GP are the only exclusion criteria.

## 4.6. STUDY VISITS AND PROCEDURES

### 4.6.1. Schedule of data collection

| **Phase** | **Stage 1**  **(6 months)** | **Stage 2**  **(18 months)** | **Stage 3**  **(3 months concurrent with Stage 2)** | **Stage 4 (Ongoing – concurrent with Stage 2)** |
| --- | --- | --- | --- | --- |
| **Establishing access to, extraction from and secure storage of de-identified data from each dataset** | **x** |  |  |  |
| **Data cleaning, merging and analysis** |  | **x** |  |  |
| **Establishment of SNOTWATCH website for public and healthcare provider access** |  |  | **x** |  |
| **Prospective data phase. NB – VAED and VEMD data will not be used for the prospective phase but may be utilised for future retrospective analyses.** |  |  |  | **x** |

# Participant withdrawals and losses to follow up

## 5.1. Withdrawal of consent

Participating sites are free to withdraw from the study at any time upon their request or the request of their legally acceptable representative. Withdrawing from the study will not affect their relationship with the Murdoch Children’s Research Institute and the Royal Children’s Hospital. The decision to withdraw the project in part or whole will be discussed with the PI. This will consider the inclusion/exclusion criteria. When a data-partner chooses to be withdrawn from the project, or is withdrawn by the research team, data collected up until that time point will be utilised in the analysis of results, unless otherwise requested by the data partner and in-line with their specific data transfer agreement.

## 5.2. Losses to follow-up

Not applicable. Research agreements between the SNOTWATCH investigators and participating Sites will delineate that that all rights, title and interest in the results of the SNOTWATCH Project which include analysis of the Data, and all associated Intellectual Property Rights (i.e., findings) will be owned solely by MCRI.

## 5.3. Replacements

Not applicable. Any new participating sites/data custodians will be subject to a HREC ethics review.

## 5.4. Study Closure

SNOTWATCH is an on-going study, therefore there is no anticipated study closure date. The recruitment of data partners for prospective data-linkage will remain open until the SNOTWATCH aims are met. The duration of this study for each participating site will be ongoing or as determined as per the research agreements. The PI will ensure that all HRECs and RGOs as well as regulatory and funding bodies have been notified of any changes.

# PRIVACY AND CONFIDENTIALITY

## 6.1. Consent

The SNOTWATCH team seek a Waiver of Consent to undertake this data linkage project in line with the National Statement section 2.3.10. which requires the following criteria to be satisfied:

| **Criteria** | **Justification** |
| --- | --- |
| 1. Involvement in the research carries no more than low risk to participants. | The study does not involve an intervention and instead helps to monitor and therefore inform how we may *prevent future* risks to participants.  This project is a data- driven epidemiological study. Retrospective data will be linked at the federally accredited Department of Health’s Azure server, Between SNOTWATCH and the Department of Health, a data-transfer system will be established that will enable near real-time geo-mapping of a range of respiratory and GI microbiology results and associated healthcare usage.  Only de-identifiable data will be available to epidemiologists and biostatisticians for analysis resulting in negligible risk to participants. |
| 1. The benefits from the research justify any risks of harm associated with not seeking consent. | SNOTWATCH’s aim is to map the typical seasonal pattern of spread of specific infections, allowing better information to be provided to community and health care providers (HCP), possibly enabling early warnings to increase preventive care such as asthma medications or vaccinations, therefor the risks outweigh the harm. |
| 1. It is impracticable to obtain consent (for example, due to the quantity, age or accessibility of records). | Yes, SNOTWATCH will utilise some of Australia’s largest datasets, thus consent is impractical. Additionally, some of the datasets that will be sourced are inherently de-identifiable so it would be impossible to contact participants. |
| 1. There is no known or likely reason for thinking that participants would not have consented if they had been asked. | The study does not involve any interventions and is focussed on geo-mapping health outcome to respiratory and GI microbiology results. The research team does not see any known or likely reason that participants would not have consented. |
| 1. There is sufficient protection of their privacy | Yes, data linkage will occur using advanced methods to protect privacy and confidentiality. The separation of roles responsible for management of identifiers and those responsible for analysing content, will protect privacy and confidentiality. |
| 1. There is an adequate plan to protect the confidentiality of data. | As above. |
| 1. In case the results have significance for the participants’ welfare there is, where practicable, a plan for making information arising from the research available to them (for example, via a disease-specific website or regional news media). | Yes, all results from SNOTWATCH will be actioned with public health as the focus. The general public and primary care professionals will also be able to view aggregate geo-mapped respiratory and GI data on the publicly available website held by the Centre of Health Analytics once the website is established. |
| 1. The possibility of commercial exploitation of derivatives of the data or tissue will not deprive the participants of any financial benefits to which they would be entitled. | No, this data will not financially impact any participants. |
| 1. The waiver is not prohibited by State, federal, or international law. | No, this waiver is not prohibited. |

## 6.2. Informed Consent Process

The entire diverse data sources used in SNOTWATCH will only be provided in de-identified format. No data provided to SNOTWATCH will be in re-identifiable format. As such, waiver of consent has been requested according to national ethics criteria. POLAR GP participating practices have an opt-in mechanism for declaring they are willing for their de-identified data (which does not identify the practice or GP) to be utilized for POLAR GP research council approved research projects. Individual patients at each practice have the ability to opt-out, in which case their data is not uploaded to the POLAR-GP data warehouse and will not be available for research.

# POTENTIAL RISKS RELATED TO STUDY CONDUCT

## 7.1. Risk to participants

There are no direct or indirect risks to participants. SNOTWATCH will utilise routinely collected data and does not involve direct patient contact, therefore greatly limiting patient risks. All analysis by the investigators will be conducted using de-identified data.

# 7.2. Benefits to participants

This study does not present any direct benefit to the participants however the study does provide an opportunity to gain a better understanding of:

- The typical seasonal pattern of spread of specific infections, allowing better information to be provided to community and health care providers (HCP), possibly enabling early warnings to increase preventive care such as asthma medications or vaccinations
- Awareness of what is circulating locally at the current time, potentially improving decisions regarding prescribing (or not prescribing) antibiotics and antivirals
- The specific impact of organisms upon health conditions and health service utilization, allowing better planning, provisioning and targeted timely communications to patients, families, and HCP

# CONFLICT OF INTEREST

There are no conflicts of interest.

# DATA AND INFORMATION MANAGEMENT

## 9.1. Publication and Presentation

SNOTWATCH aims to be a platform for the ongoing study of infectious disease epidemiology and their implications for healthcare. SNOTWATCH related research will be presented at scientific congresses in the fields of infectious diseases and microbiology, public health, emergency medicine, health informatics and general practice. Manuscripts will be submitted for publication in scientific journals in the same fields of research, with summaries available on the SNOTWATCH website.

# 9.2. Overview

The Principal Investigator is responsible for storing essential study documents relevant to data management and maintaining a site-specific record of the location(s) of the site’s data management-related Essential Documents. The Principal Investigator is responsible for maintaining adequate and accurate source documents that include all key observations on all participants at their site. Source data will be attributable, legible (including any changes or corrections), contemporaneous, original, accurate, complete, consistent, enduring and available. Changes to source data (hardcopy and electronic) must be traceable, must not obscure the original entry, and must be explained where this is necessary.

### 9.2.1. Data management

| Generation and collection – how and data be generated and collected | **Data generation (source data)**  In this study, the following types of data will be collected:   - personal identifying information (postcode of residence – Postcode, SA2 or SA3) - sensitive information including health data (respiratory multiplex PCRs and throat swab cultures; diagnosis)   All entries from participating sites and datasets (i.e., VAED) that measure outcomes will be supplied from 24^th^ July 2019 onwards. This is in addition to ongoing prospective data collection and will allow epidemiologists and SNOTWATCH investigators to analyse health outcomes with more data and generate a historical baseline and ecological analysis of health events to compare future viral respiratory illnesses and health outcomes with.  The datasets for SNOTWATCH include de-identified, electronic respiratory multiplex PCRs and throat swab cultures from each participating site. |
| --- | --- |
| Generation and collection – how and by whom will data be generated and collected  Use – how and by whom  Storage and access during the study.  Access – how and by whom, conditions under which access may be granted to others  Disclosure – the purpose for which it will be disclosed, to whom? | **Data capture methods and data use, storage, access and disclosure during the study**  Data for this study will be collected and entered using electronic data collection which will be completed by the participating pathology sites and researchers.  The following publicly available research data collection tools will be used:   - REDCap   The following licensed research data collection tools will be used:   - The Royal Children’s Hospital and the Victorian DHHS Azure secure cloud system   The following data standards will be used for coding the data:   - ICD10 for disease coding - SNOMED Codes   *Information on the data variables is located in Appendix 16.1.*  Use of the data  The data will be used for the analyses specified in the protocol and Statistical Analysis Plan. Following the completion and analysis of the study, the data will be retained long-term following the mandatory archive period for use in future research projects.  **Storage and access**  SNOTWATCH will not receive any hard copy data. Electronic data will be securely stored in MCRI's REDCap database system and in files stored in MCRI's network file servers, which are backed up nightly. Files containing private or confidential data will be stored only in locations accessible only by appropriate designated members of the research team.  REDCap is hosted on MCRI infrastructure and is subject to the same security and backup regimen as other systems (e.g. the network file servers). Data is backed up nightly to a local backup server, with a monthly backup taken to tape and stored offsite. REDCap maintains an audit trail of data create/update/delete events that is accessible to project users who are granted permission to view it. Access to REDCap will be provided via an MCRI user account or (for external collaborators) via a REDCap user account created by the MCRI system administrator. The permissions granted to each user within each REDCap project will be controlled by, and will be the responsibility of, the study team delegated this task by the Principal Investigator. REDCap has functionality that makes adding and removing users and managing user permissions straightforward. All data transmissions between users and the REDCap server are encrypted.  Authorised representatives of the sponsoring institution as well as representatives from the HREC, Research Governance Office and regulatory agencies may inspect all documents and records required to be maintained by the Investigator for the participants in this study. The study site will permit access to such records.  **Disclosure**  The study protocol, documentation, data and all other information generated will be held in strict confidence. No information concerning the study, or the data will be released to any unauthorised third party, without prior written approval of the sponsoring institution. Clinical information will not be released without written permission of the participant, except as necessary for monitoring by the HREC, Research Governance Office or regulatory agencies. |
| Methods to reduce identification of participants | Data confidentiality Participant confidentiality is strictly held in trust by the PI, participating investigators, research staff, and the MCRI and their agents. This confidentiality is extended to cover testing of microbiological samples and respiratory tests in addition to the clinical information related to diagnosis.  To preserve confidentiality and reduce the risk of identification during collection, analysis and storage of data and information, the following will be undertaken:   - The number of private/confidential variables collected for each individual has been minimised. The data collected will be limited to that required to address the primary and secondary objectives - The research team will utilise REDCap’s permission control functionality to secure pathology datasets. - Separation of the roles responsible for analysis content. - Additional information such as age, gender, or diagnosis relating to positive or negative pathology test result will be included in the data. The research team will utilise age-brackets and aggregate data to minimise the risk of re-identification. |
| Quality assurance | **Quality assurance**  Initial data cleaning, verification and auditing will be conducted at by each Pathology site, with further post-linkage cleaning and testing of linked data by the SNOTWATCH investigators at the Centre for Health Analytics. Following completion of stage 2 (See section 4.6.1), merged retrospective data will be available for analyses by SNOTWATCH collaborators following consideration by SNOTWATCH investigators to ensure non-duplication of studies. |
| Analysis – how and by whom | Data analysis will be completed by the SNOTWATCH investigator team which include epidemiologists, biostatisticians, and clinicians at the Centre for Health Analytics. See Data Analysis section 10 for more details. |
| Storage post-study ARCHIVE (after study finished and during archive period)   - how will the data be stored post-study - what is the retention period   Disposal –process for safe and secure disposal | **Archiving - Data and document retention**  This is an ongoing epidemiological analysis, as such there is no expected post-study or retention period as the data will not be archived or destroyed. However, viability of the project will be assessed every four years. In the event the project is ceased, data will be retained for 3 years.  Electronic data will be exported to an appropriate file type (e.g., csv, .xlsx) for review and will not include any identifiable details. The final dataset will be exported to an appropriate file type (e.g., csv, .xlsx) for data cleaning and statistical analysis. Only named members of the research team will have access to the databases, and this will be restricted on a role-specific basis. If data arising from this project is published in a journal, data may be made available in a data repository or archive if this is a requirement of that journal. |
| Data sharing – plans for permitting re-use of data both internal and external? | **Data sharing**  All data access or release will adhere to the internationally recognised Five Safes Framework (safe projects, safe people, safe data, safe settings and safe outputs) and the FAIR principles (Findable, Accessible, Interoperable, Reusable) (23, 24).  The data from this project will be used to inform future NHMRC and other research grant applications, to disseminate the SNOTWATCH website at state and national levels in primary health care settings. |

# 10. STUDY OVERSIGHT

## 10.1. Governance structure

A subject steering committee (SSC) will be established to provide expert advice and overall supervision and ensure that the study is conducted to the required standards. The SSC will meet at least annually, with more frequent meetings as needed.

## 10.2. Quality management, assurance and control

- The PI has responsibilities for quality management. The PI will build quality assurance (QA) into the study by developing procedures that identify, evaluate and control risk for all aspects of the study, e.g., study design, source data management and study team training, participating lab eligibility, and consent.
- The PI will also ensure that quality control (QC) procedures, which will include the checks within the data entry system and any missing data or data anomalies, are communicated to the site(s) for clarification/resolution.
- QC activities will be undertaken by Centre of Health Analytics and will be overseen by the PI, who will check that the study is conducted, and that data are generated, documented (recorded), and reported are in compliance with the protocol and applicable regulatory requirements.
- In addition, each site will perform internal QC activities to check that study conduct, data, and essential documentation follows the protocol and applicable regulatory requirements.

# 11. DATA ANALYSIS

## 11.1. Sample size and statistical power

In line with the SNOTWATCH aims the focus will be on attaining retrospective data within a timeframe of 7-10 years dating from the enrolment of each participating site/dataset. SNOTWATCH prospective phase is an ongoing epidemiological/ecological analysis which will map viral respiratory and GI microbiology results in real-time to inform health outcomes registered in Victorian health datasets. For instance, most of the results will come from individuals who have hospital records located in either VAED or VEMD datasets that can be linked to analyse respiratory outcomes. A smaller subset of individuals will have primary care records located in POLAR, and BloodSTAR (National Blood Authority) that can be linked to further analyse rare paediatric health outcomes.

The investigators aim to collaborate with other Australian jurisdictions in the future to increase the sample size. SNOTWATCH will involve a much larger population than any other viral respiratory disease surveillance method in Australia. The large sample size will have significant statistical advantages including increased precision of estimates and lower random error.

## 11.2. Statistical methods

Descriptive statistics will be used to summarise data from each of the datasets.

Retrospective data will be combined to enable statistical analysis, using platforms such Microsoft PowerBI for visual presentation, ArcGIS software for mapping purposes, SaTScan software to assess spatio-temporal correlation and R (coding language) will be utilised through R studio to perform further statistical analysis and compare statistical methods. Natural language processing of free text data such as ED triage presentation data will be assessed against presenting complaint notes from the 3 ED Departmental software records to improve the interpretation of triage text.

Due to the complexity of the multiple data sets’ potential interactions under the SNOTWATCH project, specific statistical analysis techniques will be developed and applied between studies during the prospective and retrospective phases. A comparison between the various techniques applied to the respective phases will be conducted to determine which method is most appropriate for the prospective arm.

Health outcomes will not be traceable to the individual and only population-level healthcare recommendations can be made. All analyses are routinely conducted by skilled epidemiologists and biostatisticians and interpreted with the aid of statistical measures and clinical input. Full statistical methods will be described in a separate Statistical Analysis Plan that will continue to be updated.

## 11.3. Population to be analysed

Microbiology data from participating pathology services is aimed to encompass an unknown proportion representative of the Victorian population. VAED and VEMD captures more than 95% of all (public and private) episodes of care in Victoria.

### 11.3.1. Handling of missing data

If data is missing from a particular time frame or postcode, that block of time/ postcode will be removed from analysis.

### 11.3.2. Data limitations

Some variables that will be analysed may be influenced by coding models such as ICD-10 codes. These variables typically have high completeness but may be subject to bias in which codes are recorded. As healthcare provider behavior may change over time in response to knowledge, guidelines, or funding models, this may affect the consistency of the variables used over time and erroneously imply an increase or decrease of a certain presentation. Additionally, some variables only require one entry, so the full clinical picture of a patient’s presentation is not captured. The study investigators maintain close relationships with medical staff collecting data at the hospital and primary care sites. Changes to the use of variables will be queried when a sudden change in epidemiological patterns become evident.

Patient or population factors may lead to missing data which may result in systematic bias in the reported clinical results (1). Epidemiologists and biostatisticians will refer to this bias when interpreting the results.

The Centre for Health Analytics has a team of Natural Language Processing (NLP) specialists who are working on sophisticated methods to pull out key medical information while ignoring potentially identifiable patient information. Identifiable fields will not yet be collected but may be added in the future when NLP technology advances to a level where patient privacy can be guaranteed (subject to agreement by the SSC, all data holders and the ethics committee).

# 12. ETHICS AND DISSEMINATION

## 12. 1 Research Ethics Approval & Local Governance Authorisation

The study will be conducted in full conformance with principles of the “Declaration of Helsinki”, Good Clinical Practice (GCP), the National Statement on Ethical Conduct in Human Research (NHMRC, 2007), Australian Code for the Responsible Conduct of Research (2007) and within the laws and regulations Australia.

Ethical approval will be sought from the Monash Centre for Health Research and Implementation (MCHRI). This protocol and any subsequent amendments will be reviewed and approved by the human research ethics committee (HREC) prior to commencing the research. A letter of protocol approval by HREC will be obtained prior to the commencement of the study, as well as approval for other study documents requiring HREC review.

Site-specific assessment, registration and authorisation will be requested from all database holders. A letter of authorisation will be obtained from the RGO prior to the commencement of the research at that institution. Institutional governance authorisation for any subsequent HREC-approved amendments will be obtained prior to implementation at each site.

## 12.2. Amendments to the protocol

This study will be conducted in compliance with the current version of the protocol. Any change to the protocol document or Informed Consent Form that affects the scientific intent, study design, or may affect our data suppliers willingness to continue participation in the study is considered an amendment, and therefore will be written and filed as an amendment to this protocol and/or informed consent form. All such amendments will be submitted to the HREC, for approval prior to being implemented.

## 12.3. Protocol deviations and serious breaches

A protocol deviation is any noncompliance with the study protocol, GCP, or HREC requirements. The noncompliance may be either on the part of the participant, the investigator, or the study site staff.

The following steps will be undertaken when a deviation/breach is faced:

- Where non-compliance significantly affects reliability of results, a root cause analysis will be undertaken, and a corrective and preventative action plan will be prepared.
- Where protocol deviations or serious breaches identify protocol-related issues, the protocol will be reviewed and, where indicated, amended.

The principal investigator will use continuous vigilance to identify and report deviations within 72 hours of identification of the protocol deviation. All deviations will be addressed in study source documents and will be reported to the approving HREC(s) and site Research Governance Officer(s).

# 13. DATA SUPPLIER REIMBURSEMENT

Not applicable. All data extraction costs will be facilitated by the PI via the SNOTWATCH budget.

# 14. FINANCIAL DISCLOSURE AND CONFLICTS OF INTEREST

There are no financial conflicts of interest. PI Jim Buttery is currently seconded to Department of Health Victoria part time ending 31/12/2021.

# 15. PUBLICATION AND INTELLECTUAL PROPERTY PLAN

## 15.1. Dissemination and translation plan

No individual-level data will be released from the SNOTWATCH project from the Centre of Health Analytics (MCRI).

Key findings will be recommended for inclusion in professional development offerings for healthcare providers. Abstracts will be offered for presentations at major public health conferences where viral respiratory viruses are relevant including the Australian Public Health Conference, The World Congress of Epidemiology and World Paediatrics conference. Papers will be published in referenced in public health, infectious disease, or paediatric journals.

## 15.2. Dissemination of results to participants

All findings will be made available to the general public and health professionals via the planned developed SNOTWATCH public-facing website.

Following analysis, article publications and development of the SNOTWATCH website, the following will be made available long-term for use by future researchers from a recognised research institution whose proposed use of the data has been ethically reviewed and approved by an independent committee and who accept MCRI’s conditions for access:

- Aggregated, de-identified data that underlie the results may be shared with our data partners following Material/Data Transfer Agreements (text, tables, figures and appendices)
- Analysis of the retrospective, de-identified and aggregated datasets will be published in an academic peer-reviewed journals. Secondary analyses of the retrospective and on-going prospective data linkage may result in additional scientific publications.

The findings from SNOTWATCH will be presented at national and international conferences for relevant fields of research (i.e., epidemiology and infectious diseases etc.). A final report including the key study findings and recommended health policy reform for future translation will be provided to the funders and data-partners. A lay summary will be provided to a data-partners, as well as presented in the publicly facing SNOTWATCH website to enable findings to be communicated to interested members of the public, as well as public health professionals.

## 15.3. Intellectual property

The Executive Committee will be responsible for developing publication procedures and resolving authorship issues.

# APPENDIX

## 16.1. Requested variables from each dataset

| **State or Territory Dataset** | |
| --- | --- |
| **Name of state or territory dataset:** | Victorian Emergency Minimum Dataset |
| **Data custodian:** | Department of Health (Victoria) |
| **Data requested for the period** | Ongoing |
| **Requested variables** | |
| ICD10AM Discharge Code/s | Age band according to VAED criteria |
| Patient residence location by statistical area (SA2 or SA3) | Date of presentation |
| Gender |  |

| **State or Territory Dataset** | |
| --- | --- |
| **Name of state or territory dataset:** | Victorian Admitted Episodes Dataset |
| **Data custodian:** | Department of Health (Victoria) |
| **Data requested for the period** | Ongoing |
| **Requested variables** | |
| ICD10AM Discharge Code/s | Age (Years, or if less than one year of age: <6m, 6m-11m) |
| Patient residence location by statistical area (SA2 or SA3) | Date of admission |
| Gender |  |

| **State or Territory Dataset** | |
| --- | --- |
| **Name of state or territory dataset:** | Tele-health Triage Calls |
| **Data custodian:** | Ambulance Victoria |
| **Data requested for the period** | Ongoing |
| **Requested variables** | |
| Age (Years, or if less than one year of age: <6m, 6m-11m) | Primary complaint/reason or call category |
| Caller location by postcode or statistical area 2 or 3 | Date of call |
| Gender of consumer call | Free text from primary complaint notes |
| Presence of specific categorical recorded solicited symptoms (e.g., fever/respiratory distress/ chest pain) | Categorical nature of advice given (home care/GP phone transfer/ advice to see GP/advice to attend ED/Ambulance called) |

| **State or Territory Dataset** | |
| --- | --- |
| **Name of state or territory dataset:** | Ambulance attendances |
| **Data custodian:** | Ambulance Victoria |
| **Data requested for the period** | Ongoing |
| **Requested variables** | |
| Diagnosis code/s or categories | Age (Years, or if less than one year of age: <6m, 6m-11m) |
| Patient residence location by statistical area or postcode | Date of presentation |
| Gender | Free text entry regarding presenting complaint |
| Presentation classification | Treatment given |

| **State or Territory Dataset** | |
| --- | --- |
| **Name of state or territory dataset:** | Tele-health Triage Calls |
| **Data custodian:** | Nurse on Call |
| **Data requested for the period** | Ongoing |
| **Requested variables** | |
| Primary complain/reason for call category | Age of consumer call in years (or if less than one year of age: <6m, 6m-11m) |
| Caller location by postcode or statistical area 2 or 3 | Date of call |
| Gender of consumer | Free text from primary complaint notes |
| Presence of specific categorical recorded solicited symptoms (e.g., fever/respiratory distress/ chest pain | Categorical nature of advice given (home care/GP phone transfer/ advice to see GP/advice to attend ED/Ambulance called) |

| **State or Territory Dataset** | |
| --- | --- |
| **Name of state or territory dataset:** | Tele-health Triage Calls |
| **Data custodian:** | Nurse on Call |
| **Data requested for the period** | Ongoing |
| **Requested variables** | |
| Primary complain/reason for call category | Age of consumer call in years (or if less than one year of age: <6m, 6m-11m) |
| Caller location by postcode or statistical area 2 or 3 | Date of call |
| Gender of consumer | Free text from primary complaint notes |
| Presence of specific categorical recorded solicited symptoms (e.g., fever/respiratory distress/ chest pain | Categorical nature of advice given (home care/GP phone transfer/ advice to see GP/advice to attend ED/Ambulance called) |

| **State or Territory Dataset** | |
| --- | --- |
| **Name of state or territory dataset:** | BloodSTAR |
| **Data custodian:** | National Blood Authority |
| **Data requested for the period** | Ongoing |
| **Requested variables** | |
| Intravenous immunoglobulin usage | Kawasaki IVIg |
|  |  |

| **State or Territory Dataset** | |
| --- | --- |
| **Name of state or territory dataset:** | Rapid Health Emergency Medicine Monitoring System (RHEMMS) |
| **Data custodian:** | Department of Health and Human Services |
| **Data requested for the period** | Ongoing |
| **Requested variables** | |
| Discharge diagnosis code/s ICD10AM | Age (Years, or if less than one year of age: <6m, 6m-11m) |
| Patient residence location by postcode | Date of presentation |
| Gender | Free text entry from triage staff regarding presentation |
| Presentation classification |  |

| **Private Dataset** | |
| --- | --- |
| **Name of state or territory dataset:** | POLAR |
| **Data custodian:** | Outcome Health |
| **Data requested for the period** | Ongoing |
| **Requested variables** | |
| Presenting complaint/reason | Age (Years, or if less than one year of age: <6m, 6m-11m) |
| Patient residence location by postcode | Date of presentation |
| Gender | Free text from presenting complaint notes |
| Presentation SNOWMED classifications | Investigations requested |
| Any prescriptions provided |  |

| **Private Dataset** | |
| --- | --- |
| **Name of state or territory dataset:** | Cerner |
| **Data custodian:** | Alfred Health |
| **Data requested for the period** | Ongoing |
| **Requested variables** | |
| Patient - patient status | Date of presentation |
| Age (Years, or if less than one year of age: <6m, 6m-11m) | Diagnosis codes (ICD-10-AM) |
| Residential Address (linkage purposes only) | Post code (SA2) |
| Diagnosis - Diagnosis field (SNOMED where possible otherwise free text) | State |
| Length of Stay in ED | Free text entry from triage staff |
| Investigations performed | Free text entry from complaint notes |
| Gender |  |

| **Private Dataset** | |
| --- | --- |
| **Name of state or territory dataset:** | Symphony |
| **Data custodian:** | Monash Health |
| **Data requested for the period** | Ongoing |
| **Requested variables** | |
| Patient - patient status | Date of presentation |
| Age (Years, or if less than one year of age: <6m, 6m-11m) | Diagnosis codes (ICD-10-AM) |
| Residential Address (linkage purposes only) | Post code (SA2) |
| Diagnosis - Diagnosis field (SNOMED where possible otherwise free text) | State |
| Length of Stay in ED | Free text entry from triage staff |
| Investigations performed | Free text entry from complaint notes |
| Gender |  |

| **Private Dataset** | |
| --- | --- |
| **Name of state or territory dataset:** | Epic |
| **Data custodian:** | Royal Children’s Hospital |
| **Data requested for the period** | Ongoing |
| **Requested variables** | |
| Patient - patient status | Date of presentation |
| Age (Years, or if less than one year of age: <6m, 6m-11m) | Diagnosis codes (ICD-10-AM) |
| Residential Address (linkage purposes only) | Post code (SA2) |
| Diagnosis - Diagnosis field (SNOMED where possible otherwise free text) | State |
| Length of Stay in ED | Free text entry from triage staff |
| Investigations performed | Free text entry from complaint notes |
| Gender |  |
